# Quantifying resolution in pink-beam dark-field X-ray microscopy: experiments and simulations

**DOI:** 10.1107/S1600576726005200

**Published:** 2026-07-07

**Authors:** M. La Bella, H. F. Poulsen, S. Staeck, N. A. Henningsson, M. P. Kabukcuoglu, C. Detlefs, C. Yildirim

**Affiliations:** a Technical University of Denmark, 2800 Kgs Lyngby, Denmark; b European Synchrotron Radiation Facility, 38043 Grenoble, France; c Institute for Photon Science and Synchrotron Radiation (IPS), Karlsruhe Institute of Technology (KIT), 76344 Eggenstein-Leopoldshafen, Germany; d Leibniz-Institut für Kristallzüchtung (IKZ), 12489 Berlin, Germany; Diamond Light Source, United Kingdom

**Keywords:** dark-field X-ray microscopy, pink-beam illumination, compound refractive lenses, orientation mapping

## Abstract

Pink-beam dark-field X-ray microscopy (pDFXM) is a powerful emerging technique for time-resolved studies of microstructure and strain evolution in bulk crystalline materials. In this work, we systematically assess the performance of pDFXM relative to monochromatic DFXM when using a compound refractive lens as the objective. Analytical expressions for the spatial and angular resolution are derived and compared with numerical simulations based on geometrical optics and experimental data. The pink-beam configuration provides an increased diffraction intensity depending on the deformation state of the sample, accompanied by a general tenfold degradation in angular resolution along the rocking and axial strain (longitudinal) directions. This trade-off is disadvantageous for axial strain mapping but can be advantageous in cases where integrated intensities are needed. For a perfect crystal under parallel illumination with a pink beam, our results show that chromatic aberration is absent, whereas under condensed illumination it becomes significant. The aberration is shown to depend strongly on the local distortion of the crystal. Weak-beam imaging conditions, such as those required for resolving dislocations, are shown to remain feasible under pink-beam operation and may even provide an improved signal-to-noise ratio. The higher incident flux, enhanced by nearly two orders of magnitude, results in a ∼30× increase in diffracted intensity; beam heating effects are quantified and implications for optimized scanning protocols are discussed.

## Abstract 2

A quantitative comparison between pink-beam and monochromatic dark-field X-ray microscopy (DFXM) shows that pink-beam DFXM offers up to two orders of magnitude higher flux and improved signal-to-noise ratio for time-resolved imaging, at the expense of an approximately tenfold loss in angular resolution. The results establish performance criteria and experimental strategies for optimizing pink-beam DFXM in studies of microstructure and strain evolution in crystalline materials.

## 1. Introduction

Dark-field X-ray microscopy (DFXM) has emerged as a powerful full-field imaging technique for the non-destructive 3D mapping of strain, orientation and defect structures within bulk crystalline materials (Simons *et al.*, 2015 ▸; Poulsen *et al.*, 2017 ▸). By selectively imaging Bragg-diffracted X-rays, DFXM enables high-resolution characterization of microstructural features such as dislocations, domain structures and strain gradients with sub-micrometre spatial resolution and milliradian angular sensitivity. The technique has been instrumental in studying a wide range of materials science phenomena, including plastic deformation, nucleation and growth, structural and magnetic phase transformations, ferroelectric behavior, biominerals, and imaging of acoustic waves (Ahl *et al.*, 2017 ▸; Simons *et al.*, 2018 ▸; Cook *et al.*, 2018 ▸; Mavrikakis *et al.*, 2019 ▸; Bucsek *et al.*, 2019 ▸; Dresselhaus-Marais *et al.*, 2021 ▸; Yildirim *et al.*, 2023 ▸; Holstad *et al.*, 2023 ▸; Lee *et al.*, 2025 ▸; Gürsoy *et al.*, 2025 ▸; Zelenika *et al.*, 2025 ▸; Zhou *et al.*, 2025 ▸). DFXM is often applied in combination with other diffraction imaging techniques, allowing multiscale characterization of hierarchically organized materials (Gustafson *et al.*, 2020 ▸; Gustafson *et al.*, 2023 ▸; Chen *et al.*, 2023 ▸; Lee *et al.*, 2024 ▸; Shukla *et al.*, 2025 ▸).

The first implementations of DFXM have relied on monochromatic X-ray beams, providing well-defined reciprocal space selectivity and high angular resolution. This makes monochromatic DFXM particularly suitable for resolving subtle orientation variations in bulk crystalline materials. However, the technique’s reliance on a narrow-bandwidth X-ray beam results in inherently low photon flux, which leads to long exposure times and limits its applicability for materials with a weak diffraction signal. Moreover, highly deformed materials with large intra-grain orientation spreads often require impractically long acquisition times, restricting real-time and high-throughput studies. These time constraints and limitations due to sample deformation also pose a major challenge for methods aiming to combine multiple Bragg reflections to reconstruct embedded tensor fields with DFXM (Henningsson *et al.*, 2025 ▸; Detlefs *et al.*, 2025 ▸). To address these limitations, pink-beam DFXM (pDFXM) was developed at the new beamline ID03 at the European Synchrotron Radiation Facility, ESRF (Isern *et al.*, 2025 ▸). The pink beam is obtained by using a double multilayer monochromator instead of the classical double crystal Si monochromator. The pink beam was demonstrated to provide a 27-fold increase in intensities (Yildirim *et al.*, 2025 ▸).

However, the use of a broadened energy bandwidth may introduce chromatic aberration, deteriorating spatial resolution, similar to what has been observed in bright-field X-ray microscopy (Falch *et al.*, 2016 ▸). Likewise, the reciprocal space resolution is impacted, altering strain sensitivity and dislocation contrast. The increased bandwidth also affects the effective angular selectivity, leading to potential trade-offs between improved photon efficiency and resolution degradation. Furthermore, the higher flux associated with pDFXM raises concerns regarding beam heating and, for some materials, beam-induced radiation damage.

In this study, we introduce a geometrical-optics-based formalism for pDFXM to describe both direct and reciprocal space resolutions. Then, we present a systematic comparison of monochromatic and pink-beam DFXM, examining their respective resolution functions. We generalize existing geometrical optics theory (Poulsen *et al.*, 2017 ▸) to the pink-beam case, providing closed expressions for key microscope characteristics. Next, we combine experimental measurements with numerical simulations to quantify the effects of beam divergence and energy bandwidth broadening on spatial resolution, angular selectivity and weak-beam contrast, *i.e.* the contrast arising from diffraction conditions far from the Bragg peak, at the tails of the rocking curve. Finally, we show the effect of pink-beam operations on the temperature rise of a specific specimen, providing thermal decay times and the impact on measurement protocols.

## 2. Dark-field X-ray microscopy geometry

**Figure 1:**
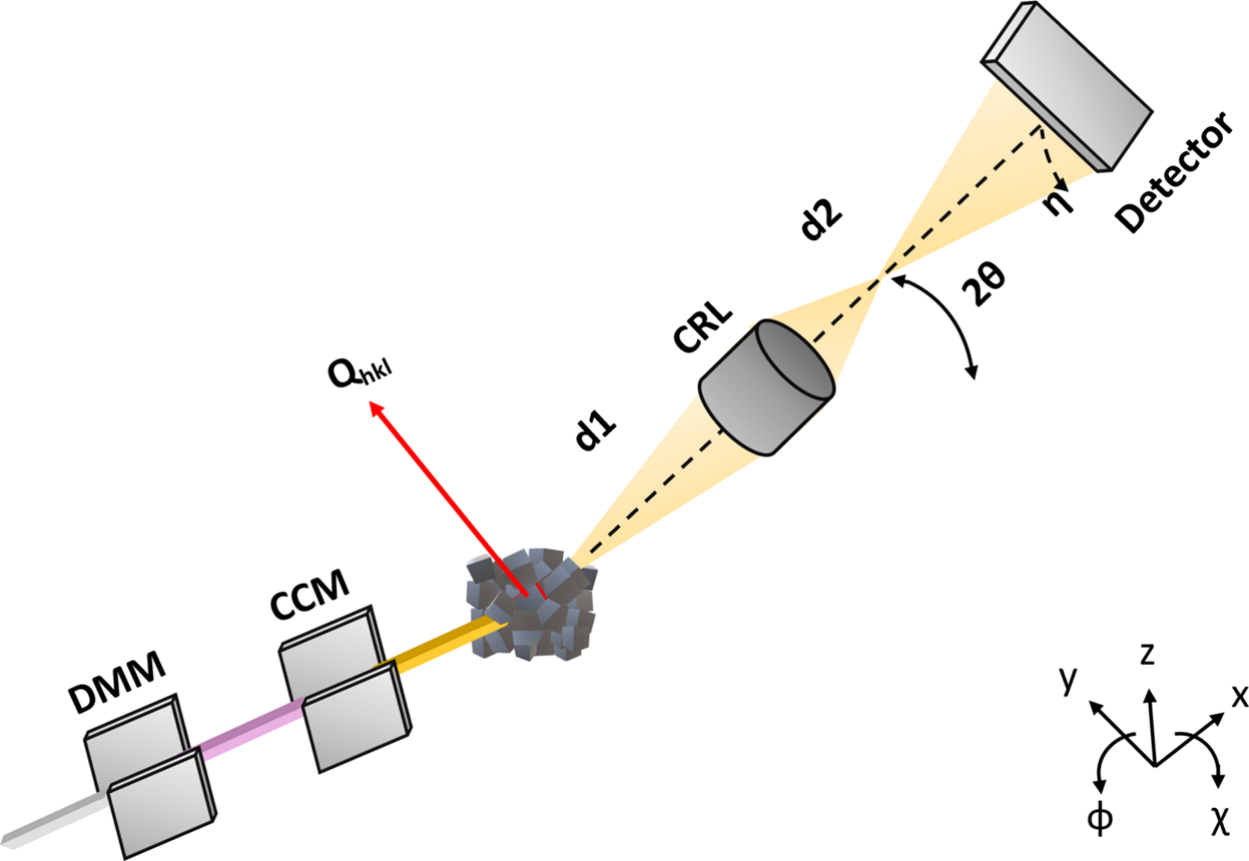
The DFXM setup for monochromatic illumination. The incident beam is shaped by the double multilayer monochromator (DMM) and then by the channel-cut monochromator (CCM). The objective is a CRL. This is inserted in the line of the diffracted beam, characterized by angles 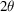 and η. The optical axis of the objective is shown as a dashed line with indications of the sample-to-entry-of-objective distance, 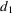, and the exit-of-objective-to-detector distance, 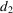.

Fig. 1 ▸ outlines the geometry of DFXM. The energy of the incident beam is defined by either a channel-cut crystal monochromator—in the following termed a monochromatic beam—or a double multilayer monochromator—a pink beam. The energy bandwidth, 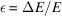 with *E* being the energy in eV, is approximately 10^−4^ and 10^−2^ in the respective cases. The incident beam can be focused vertically using a condenser to form a line beam or left unfocused to produce a box beam, *i.e.* a quasi-parallel beam with a rectangular cross-section. The incident beam has a divergence characterized by an FWHM of Δ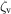 and Δ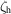 in the vertical and horizontal directions, respectively (Poulsen *et al.*, 2017 ▸).

The sample can be either a single crystal or a polycrystalline aggregate. In both cases the goniometer movements are used to bring a specific grain or domain into the Laue conditions. The reflection of interest is indicated with a diffraction vector 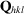. It is defined by the diffraction angle 2θ and an azimuthal angle η (Poulsen *et al.*, 2017 ▸). In the following we will assume a vertical scattering geometry: 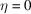.

In a typical DFXM experiment, an objective is placed in the diffracted beam, such that the optical axis of the objective intersects with the pivoting point of the goniometer and a 2D detector. The objective magnifies diffraction contrast from the sample plane into an inverted 2D image on the detector. In this paper, the X-ray objective will be a compound refractive lens (CRL; Snigirev *et al.*, 1996 ▸) comprising *N* identical parabolic lenslets, with a radius of curvature at the apex *R* and a distance between neighboring lenslets of *T*. The distance from the sample to the CRL entrance is 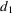, and the distance from the CRL exit to the detector is 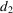. The total optical path length is 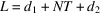, where *NT* is the combined thickness of the lens stack. The resulting magnification is 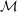.

The sample is mounted in a goniometer that enables rotations around multiple axes. Shown in Fig. 1 ▸ are the two orthogonal tilts χ and ϕ. Scanning ϕ, χ and 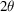, a motion of CRL and detector together, corresponds to probing contrast by varying 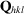 in three orthogonal directions. These scans are known as rocking, rolling and axial strain scans, respectively. A more complete geometrical description is provided by Poulsen *et al.* (2017 ▸) and Poulsen *et al.* (2021 ▸). The DFXM goniometer may include additional degrees of freedom. Details of the dedicated DFXM setup at beamline ID03 of the ESRF, used in this work, are given by Isern *et al.* (2025 ▸).

## 3. Geometrical optics formalism for a pink beam

A formalism for the main properties of the microscope was developed on the basis of geometrical optics by Simons *et al.* (2017 ▸), Poulsen *et al.* (2017 ▸) and Poulsen *et al.* (2018 ▸). We here summarize key equations for the monochromatic-beam case and generalize these to the use of a pink beam.

### 3.1. Focal length, numerical aperture and field of view

For brevity, we introduce the shorthand 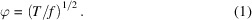
In monochromatic-beam conditions, the focal length of one lenslet is 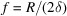, where the refractive index decrement 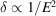. For *N* identical lenslets, the focal length becomes 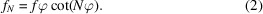
In a pink beam, 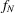 varies with energy, leading to a relative spread of (thick or thin lens limit) 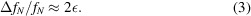
In general, the average 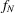 is linked to the magnification of the objective 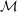 by 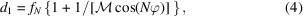

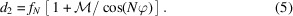
Since the detector and CRL positions are fixed, the variation in focal length described by equation (3) causes each energy component to produce a slightly different magnification. From equations (3) and (5), the image plane shifts along the optical axis by approximately 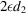 across the pink-beam energy range, corresponding to a relative change in magnification of 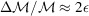.

For the numerical aperture, NA, we shall consider *the opaque lens case* where the effective aperture is dominated by attenuation in the parabolic part of the lens, and not the physical aperture. For a monochromatic beam, the FWHM of the attenuation distribution in the on-axis case is (Poulsen *et al.*, 2017 ▸)
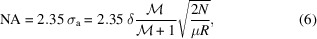
with μ being the linear attenuation coefficient and σ_a_ the pupil function, leading to a Gaussian angular acceptance and thus a numerical aperture with r.m.s. width σ_a_. The variation within an energy spread of 1% is negligible; hence this equation is also valid for the pink-beam case. The same applies for the field of view.

### 3.2. Direct space resolution

The depth of field, 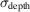, for monochromatic-beam DFXM is given by the combination of a wave optics and a geometrical optics term, defined by the resolution in the imaging plane 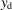 (Poulsen *et al.*, 2017 ▸): 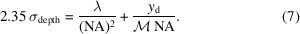
This appears to be essentially constant over a 1% energy band. The *chromatic aberration* is a concern for pink-beam operation, as the focal length and magnification vary with energy as discussed in Section 3.1 (Poulsen *et al.*, 2017 ▸). This is expected to impact the data in several ways. Firstly, for line beam operation the vertical beam height will increase by approximately 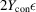. Here, 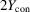 is the effective physical aperture of the condenser—which may be defined by an upstream aperture. For typical operation at ID03, 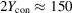
 µm and hence the beam height becomes 1.5 µm for 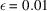.

Secondly, in relation to the objective, in bright-field microscopy there will be a lateral/transverse chromatic aberration—a smear in the radial direction *r*—in the image, also known as the transverse chromatic aberration. This effect is described theoretically and demonstrated experimentally by Falch *et al.* (2016 ▸). Here, it is also shown that one can mitigate the effect by focusing the beam from the condenser onto the plane of the objective. However, this elegant solution and the underlying formalism do not apply to dark-field microscopy. Analytical expressions for this case are complicated [see Simons *et al.* (2017 ▸)]. In this paper we will instead provide numerical results based on DFXM ray tracing (see Section 4.2).

### 3.3. Reciprocal space and strain resolution

The angular resolution of DFXM is essentially determined by properties of the incident beam and the diffracted beam (including the objective). The Darwin width intrinsic to the sample can typically be neglected.

Before entering into the formalism for the resolution function, it is useful to define three coordinate systems. Following Poulsen *et al.* (2021 ▸) we adopt the following:

(1) *The laboratory system.* This is described by the axes (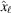, 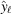, 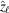) and is used to express the properties of the incident beam.

(2) *The imaging system.* This is used to describe the diffracted beam. It has the optical axis of the objective as its *x* axis and is related to the laboratory system by a rotation around the *y* axis by 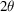. The corresponding reciprocal space axes are defined as (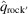, 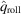, 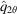).

(3) *The crystal system.* This is used to describe diffraction properties. It is defined as having the scattering vector 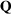 along the **z** direction and is described by the axes (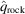, 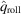, 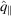) in reciprocal space. It is related to the laboratory system by a rotation around the *y* axis by θ.

The reciprocal space resolution at the intersection between the optical axis and the sample plane is described in the following. The projections of resolution function onto the three axes of *the crystal system* are (Poulsen *et al.*, 2017 ▸)
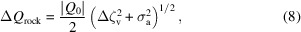

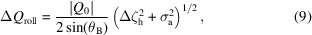

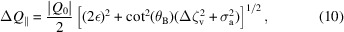
with 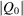 being the length of the nominal diffraction vector and 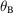 being the Bragg angle. It is evident that a change in the energy bandwidth only affects 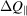. In the *imaging coordinate system*, on the other hand, due to the rotation of θ broadening will appear in both the rocking and the 2θ direction. Since the range of the reciprocal space probed is small, it is convenient to work with normalized diffraction vectors (strain units) (Poulsen *et al.*, 2021 ▸): 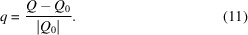


### 3.4. Back focal plane

As for any classical microscope the objective in DFXM is associated with a Fourier plane, the back focal plane, BFP. This is perpendicular to the optical axis. For completeness we discuss the properties in this plane in relation to an increased bandwidth.

Let the positions in the BFP be parameterized by 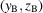. For a monochromatic and parallel beam the positions are linearly related to the angular deviations from the optical axis. Expressed in terms of the strain in the rolling and 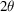 directions we have the relations (Poulsen *et al.*, 2018 ▸)
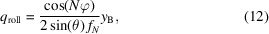

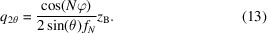
These relations become more complicated when switching to a non-monochromatic and divergent pink beam (Poulsen *et al.*, 2017 ▸): 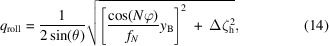

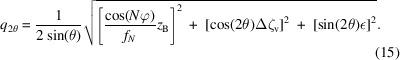
In the monochromatic-beam case, the relatively poor axial strain resolution in DFXM can be improved by inserting an aperture or a knife-edge in the BFP. It appears from equations (14) and (15) that in the pink-beam case the strain resolution in the 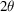 direction is ultimately limited by the energy bandwidth. Hence, the achievable strain resolutions in the 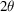 direction are about 10^−4^ and 10^−2^, for monochromatic and pink beams, respectively.

## 4. Simulations

Following previous work (Poulsen *et al.*, 2017 ▸; Borgi *et al.*, 2024 ▸) we use Monte Carlo simulations to provide maps of the on-axis (normalized) reciprocal space resolution function for both pink and monochromatic beams. Next, we use a ray-tracing simulation approach to provide insights into chromatic aberration. Two model systems are used throughout the simulations and experiments: a thin-film BiFeO_3_ sample providing the 002 reflection and a bulk Si wafer diffracting on the (220) planes. These were chosen as representative of two common use cases: a weakly scattering thin film and a strongly diffracting bulk crystal, respectively. Sample details, together with the experimental configurations used in the simulations (including the energy spectrum of the pink beam), are given in the supplementary information (SI), Sections 1 and 5.

### 4.1. Simulations of the reciprocal space resolution function

The simulations were performed with the code presented by Borgi *et al.* (2024 ▸) and with 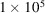 rays throughout. We assume that all properties of the microscope exhibit Gaussian distributions, defined by their r.m.s. widths.

**Figure 2:**
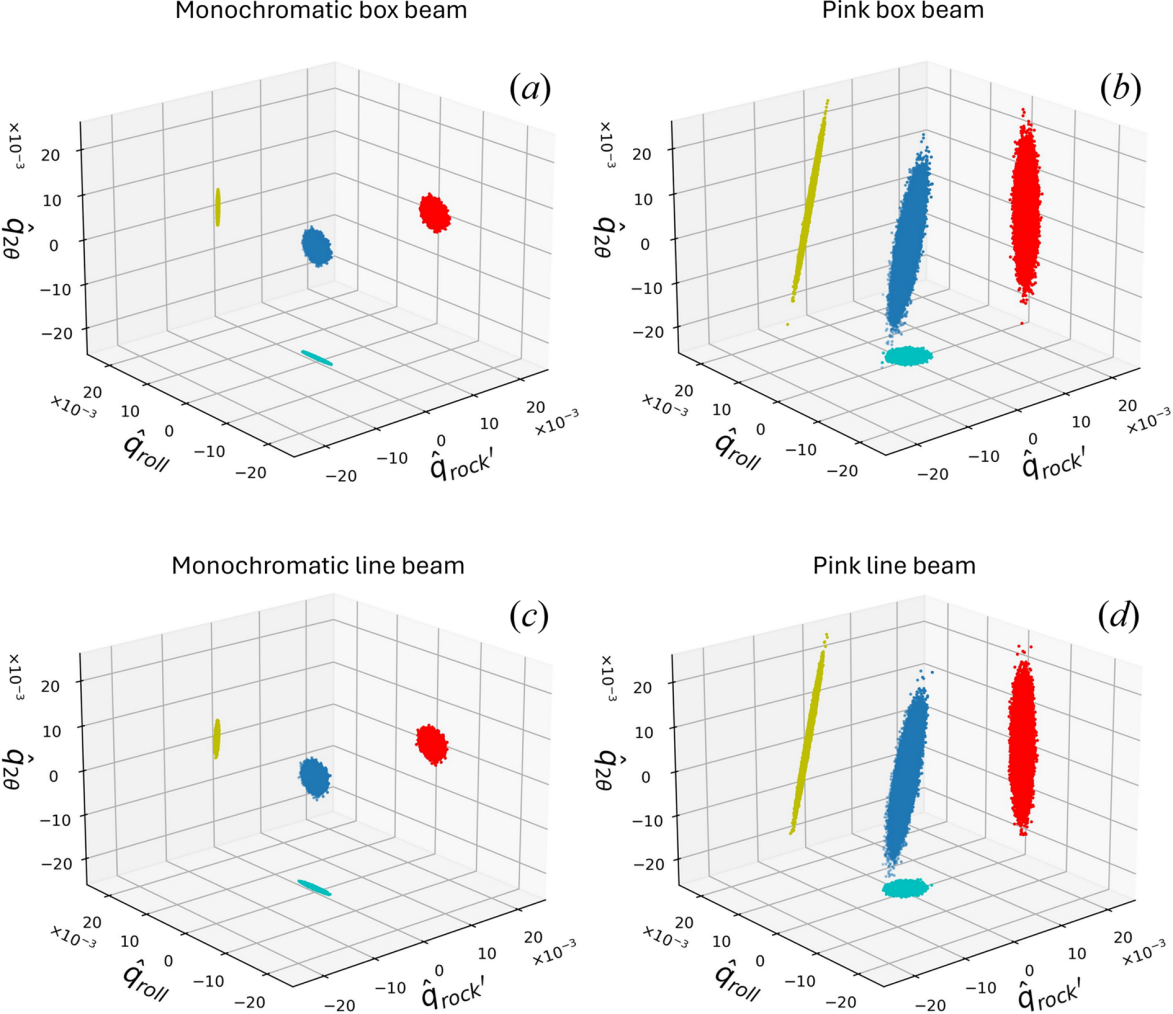
Reciprocal space resolution function for a box beam (quasi-parallel illumination) with monochromatic (*a*) and pink (*b*) beams, and line beam (condensed illumination) for monochromatic (*c*) and pink (*d*) beams. In all cases, the reciprocal space is normalized, *cf*. equation (11). The resolution function is displayed as a point cloud in three dimensions in blue, while the red, yellow and cyan point clouds are projections onto 2D planes. The three principal axes of the imaging coordinate system (*i.e.* corresponding reciprocal space axes) are defined as (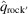, 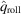, 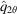), where 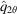 corresponds to the longitudinal (axial strain) direction.

First, we consider *the box beam case (quasi-parallel illumination)*. The resulting resolution function for the monochromatic beam in the imaging system is shown in Fig. 2 ▸(*a*). The resolution function appears to be a disk with a diameter given by the NA of the objective and with the disk axis along 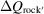. The fact that 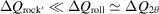 has been key to the design of DFXM weak-beam experiments.

Components of the corresponding pink-beam resolution function in the crystal system are shown Fig. 2 ▸(*b*). Essentially the figure represents a convolution of the monochromatic-beam resolution function with a Gaussian in the direction of the diffraction vector [*cf*. equation (10)]. This has several consequences for pDFXM:

(1) *Deterioration of the axial strain resolution*. Spanning a range in 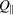 of 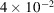 makes axial strain determination infeasible. On the other hand, pink-beam operation implies that one typically will be able to ensure integration over the entire strain profile, and hence may speed up experiments that depend on sampling the integrated intensity.

(2) *Deterioration of the rock resolution.* Weak-beam operation has benefited from the superior resolution in the direction perpendicular to the disk. In the pink-beam mode the thinnest direction in reciprocal space is ten times thicker, as was shown experimentally in our previous work (Yildirim *et al.*, 2025 ▸).

(3) *Choice of coordinate system*. The directions of the principal axes change. While the imaging system is the natural choice for monochromatic-beam operation, the crystal coordinate system is more relevant for pink-beam operation. This choice of coordinate system is conceptually more intuitive for applications.

Next, we consider *the line beam case (condensed illumination)*. The resulting resolution functions are shown in Figs. 2 ▸(*c*) and 2 ▸(*d*). While the increased divergence has some effect in the monochromatic-beam case, this contribution is completely negligible in the pink-beam case. The latter can be an advantage: it facilitates direct quantitative comparisons between line and box beam mapping.

### 4.2. Simulations of chromatic aberration

The broadened energy bandwidth in pDFXM not only impacts reciprocal space resolution, as investigated in the previous section, but also will deteriorate spatial resolution by blurring detector images in direct space. The effect is known as chromatic aberration and is well known from bright-field X-ray microscopy (Falch *et al.*, 2016 ▸). The corresponding effect in dark-field microscopy, has, to the best of our knowledge, never been studied before. Simulating these effects requires one to trace X-rays through the 3D objective lens stack as a function of wavelength. Unfortunately, previous geometrical optics work in DFXM cannot account for such ray trajectories. In frameworks such as those described by Poulsen *et al.* (2017 ▸), Borgi *et al.* (2024 ▸) and Henningsson *et al.* (2025 ▸) each point in the sample plane is approximated to deposit all diffracted photons at a corresponding, fixed detector pixel, neglecting depth-of-focus and vignetting effects. While this allows for fast simulations, where the reciprocal resolution function can be used as a look-up table for diffracted intensities, it does not allow us to simulate imaging artifacts. To progress, we have developed a new framework that traces rays in three dimensions through the objective lens stack.

After a photon has scattered off the sample, we use Snells’s law to solve a recursive set of non-linear equations that trace the ray path through the beryllium lens stack. At each lenslet, refraction from the parabolic entry and exit surface is computed, and the path length through the lens is used to attenuate the ray intensity using Beer’s law. Both of these effects are taken to be functions of the randomly sampled wavelength of the ray such that each traced ray perceives a unique refractive decrement, 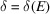, and attenuation coefficient, 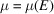. A detailed mathematical derivation of our framework is provided in SI Section 5.

Importantly, in our new framework, photons that are sampled from the direct beam undergo scattering following the Laue equations. The resulting diffracted beam is therefore a modulation of the direct beam via an interaction with the sample lattice. This makes dark-field chromatic aberrations inherently sample dependent. This is an important conclusion for pDFXM that prohibits us from generalizing a single simulation across experiments. Nevertheless, in the following we present simulations of what can be considered to be the canonical case; undeformed single crystals placed at the center of focus in the sample plane. By using experimentally observed statistics for the direct beam divergence and bandwidth these simulations quantify how severe chromatic artifacts are for practical imaging scenarios. All driving distributions are taken to be Gaussian and independent. Four distinct scenarios are examined:

(A) * Parallel and monochromatic beam*. The vertical and horizontal divergence of the direct beam were taken as 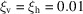
 mrad (FWHM). The energy bandwidth was taken as 0.001146 keV (standard deviation). A BiFeO_3_ 002 reflection is considered.

(B) * Condensed and monochromatic beam*. The vertical and horizontal divergence of the direct beam were taken as 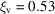
 mrad and 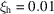
 mrad (FWHM), respectively. The energy bandwidth was taken as 0.001146 keV (standard deviation). An Si 220 reflection is considered.

(C) * Parallel and pink beam*. The vertical and horizontal divergence of the direct beam were taken as 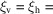 0.01 mrad (FWHM). The energy bandwidth was 0.10123 keV (standard deviation). A BiFeO_3_ 002 reflection is considered.

(D) * Condensed and pink beam*. The vertical and horizontal divergence of the direct beam were taken as 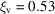
 mrad and 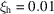
 mrad (FWHM), respectively. The energy bandwidth was 0.10123 keV (standard deviation). An Si 220 reflection is considered.

The mean energy was set to 19.1 keV and the sample-to-CRL and CRL-to-detector distances were adjusted accordingly. For parallel beams, the placement was taken to yield a 11.26 times magnification from the CRL. For the condensed cases, the CRL magnification was fixed to 15.77. The CRL featured 87 parabolic berylium lenslets with 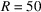
 µm, 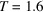
 mm and web thickness 30 µm. In all scenarios, the raw detector pixel size was 6.5 µm and the optical magnification of the detector was 10×.

**Figure 3:**
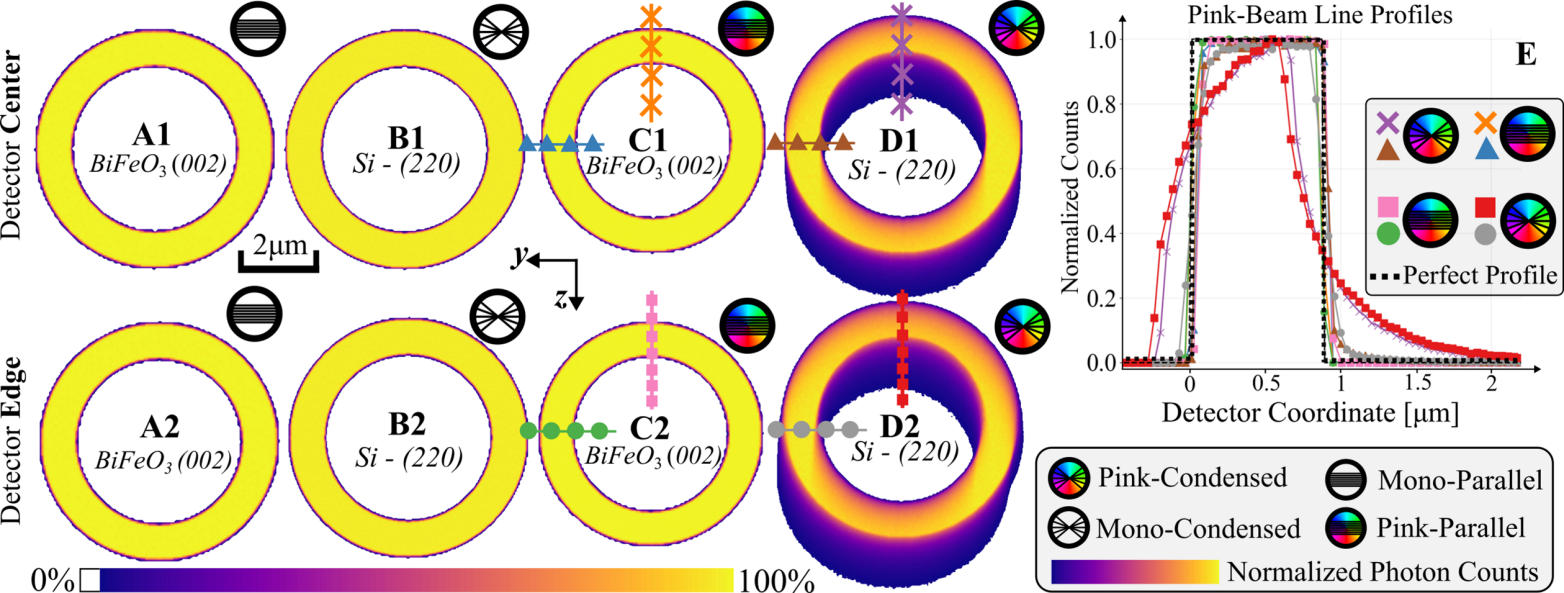
Simulated diffraction response from ring-shaped, undeformed single crystals under different beam conditions. When the beam is both pink and condensed (D1 and D2), strong chromatic aberrations appear. In A1–D1, the rings are centered on the optical axis, while in A2–D2, they diffract toward the detector edge. Cartesian line profiles across the ring edges are shown in E for all four pink-beam scenarios (C1–D2). Each line plot in panel E corresponds to an intensity profile across a ring wall as highlighted in C1–D2 with colored lines and markers. In panel E, the box-like ‘perfect profile’ (black, dotted line) represents the theoretical ring wall profile in the absence of chromatic aberration, assuming a perfect imaging system (small pixels, no point spread).

A grid of 81 ring-shaped single crystals (BiFeO_3_ 002 and Si 220 reflections) were placed in the sample plane and Monte Carlo methods were used to sample rays from the beam statistics defined though A–D. The resulting detector images for two selected rings and all beam scenarios (A–D) are presented in Fig. 3 ▸. In A1–D1, the rings centered on the optical axis have been selected, while in A2–D2, off-axis rings are shown (diffracting towards the detector edge). Each individual ring corresponds to a total of 84 million rays individually traced through the CRL lens stack.

Comparing D1 and D2 shows that chromatic aberrations depend on the offset from the optical axis. Chromatic aberrations are observed to be stronger in the vertical scattering plane (detector *z*) than in the transverse plane (detector *y*). Cartesian line profiles across the ring edges are shown in Fig. 3 ▸E for pink-beam scenarios (C1–D2). The chromatic aberrations in D1–D2 are seen to cause a 1 µm blurring, while the parallel pink-beam cases (C1–C2) show no blurring.

The lack of chromatic aberration in A1–C2 in Fig. 3 ▸ is explained by the sample acting as a monochromator, filtering the direct beam though the Bragg condition. Consequently, only when the direct beam features both a high vertical divergence and a broad energy band does chromatic aberration appear for undeformed crystals (D1–D2). In this special case, a divergent ray can diffract off the sample by being paired with an offset wavelength such that the combination again fulfills scattering conditions. Many such interactions combine into a non-negligible energy broadening being present also in the diffracted beam. Notably, the resulting statistics of the diffracted beam, *i.e.* the distribution of photon energies and propagation directions in the diffracted beam, are non-Gaussian. Consequently, dark-field aberrations do not follow the patterns predicted in bright-field microscopy (Falch *et al.*, 2016 ▸).

The fact that the sample modulates the statistics of the diffracted beam, and hence the resulting aberrations, has important implications for crystals featuring a non-perfect lattice.

In such cases, the local mosaic spread will result in diffracted beam statistics featuring significant divergence and bandwidth—even if the incident beam is parallel. In other words, the divergence of the incident pink beam in D1–D2 can be effectively replaced by a mosaic spread. It is therefore possible that for samples with moderate to large mosaic spreads the chromatic aberration can be worse than indicated in Fig. 3 ▸E. As an illustration, for crystals that partition into several near-perfect cell domains, separated by low-angle orientation boundaries, one may expect cell boundaries to exhibit stronger chromatic aberrations than cell interiors, though the extent to which the interior remains unaffected will depend on the local mosaic spread and cell size.

### 4.3. Strong- and weak-beam operation

The larger reciprocal space resolution function—as derived in Section 4.1— affects the optimal conditions for weak-beam operation. This is discussed by Borgi *et al.* (2024 ▸). Here DFXM forward projections are made of a wall of identical edge dislocations. Images are generated as function of the rocking angle, ϕ, showing little or no dislocation contrast for ϕ close to 0 (strong beam). The contrast gradually improves with increasing ϕ (weak beam) while the intensities decrease as the volume of the sample that gives rise to diffraction (close to the core of the dislocations) become smaller. Comparing such results for different energy bandwidths, it was found that the strong-beam region extends to larger 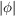 values for larger ε and that one consequently has to go to larger 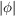 values for optimal conditions for weak-beam imaging. Larger values implies higher strain values and hence diffraction from regions closer to the core of dislocations. Hence, there are two competing effects on the signal-to-noise ratio (S/N) of dislocation imaging: larger ε implies a lager incident flux, while at the same time leading to the diffraction volumes being smaller. The simulations reported by Borgi *et al.* (2024 ▸) indicate that the increased incident flux—for the case simulated—significantly improves the counting statistics with increased energy bandwidth.

## 5. Experimental results

We conducted experiments for comparison with the analytical and numerical results presented above. Sample and configuration details are given in SI Section 1, 2 and 3. Details about BiFeO_3_ sample manufacturing are provided by Chu *et al*. (2007 ▸) and Simons *et al.* (2019 ▸).

### 5.1. Reciprocal space resolution function

To probe the rocking and rolling components, a large diamond single crystal was used as the sample, diffracting the 111 reflection.

**Figure 4:**
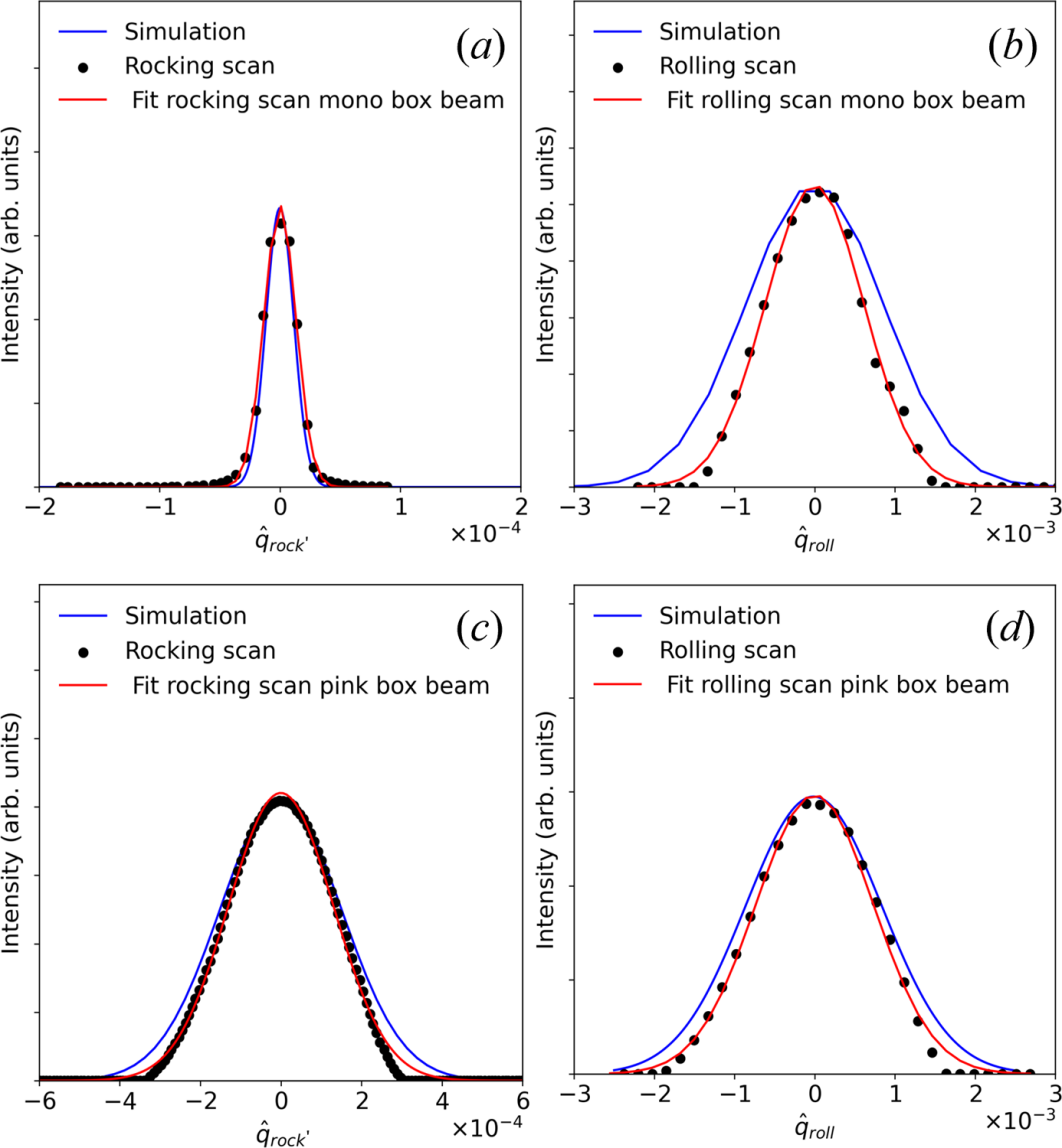
Comparison of normalized experimental data (dots) and simulations (blue curves) of the rocking and rolling direction components of the reciprocal space resolution function for the box beam case. Also shown are fits of the experimental data to Gaussian distributions (red lines). (*a*) Resolution function in 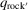 for a monochromatic beam. (*b*) Resolution function in 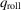 for a monochromatic beam. (*c*) Resolution function in 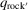 for a pink beam. (*d*) Resolution function in 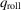 for a pink beam.

In the *box beam* case (Fig. 4 ▸), the simulations show excellent agreement with the experimental data for both directions and for both monochromatic- and pink-beam illumination. In the rolling direction, the simulations overestimate the angular spread of 0.00057 strain units; however, as shown by Poulsen *et al.* (2017 ▸), this effect arises from the scattering vector moving off the rocking curve. The corresponding FWHM values are listed in Table 1 ▸.

**Figure 5:**
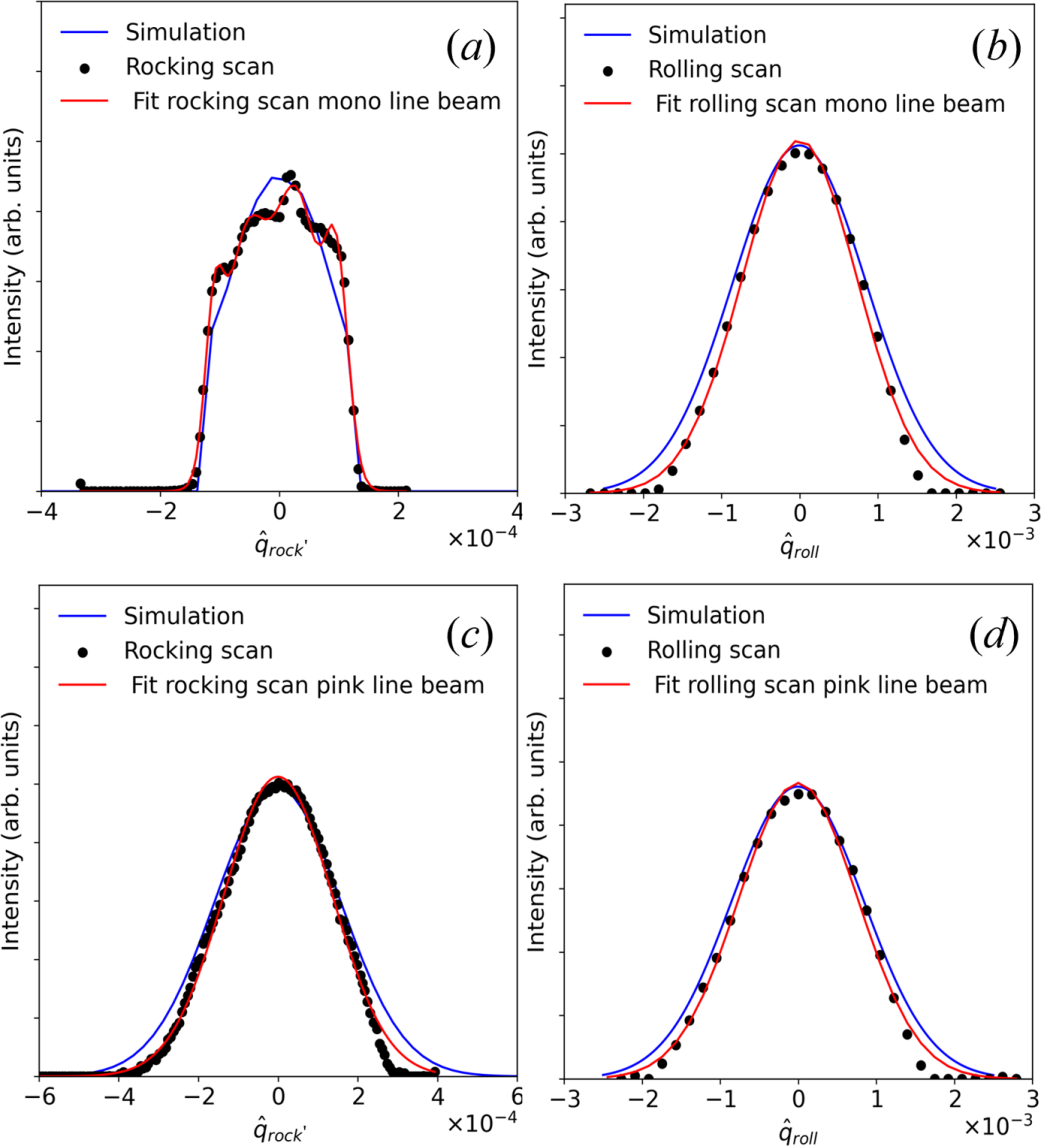
Comparison of normalized experimental data (dots) and simulations (blue curves) of the rocking and rolling direction components of the reciprocal space resolution function for the line beam case. Also shown are fits of the experimental data to Gaussian distributions (red lines). (*a*) Resolution function in 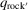 for a monochromatic beam. (*b*) Resolution function in 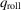 for a monochromatic beam. (*c*) Resolution function in 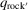 for a pink beam. (*d*) Resolution function in 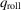 for a pink beam.

In the *line beam* case (Fig. 5 ▸), there is also good agreement between the simulated and experimental curves. For the monochromatic beam, the angular resolution function in the rocking direction [Fig. 5 ▸(*a*)] is truncated by a slit placed upstream of the condenser. Moreover, the 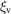 distribution was non-uniform, and both effects were accounted for in the simulations. In this case, the experimental curve was fitted with a multi-Gaussian model. The corresponding pink-beam resolution function [Fig. 5 ▸(*c*)] appears smoother due to convolution with the energy bandwidth, though a slight truncation of the tails remains. The resulting FWHM values are listed in Table 2 ▸.

**Figure 6:**
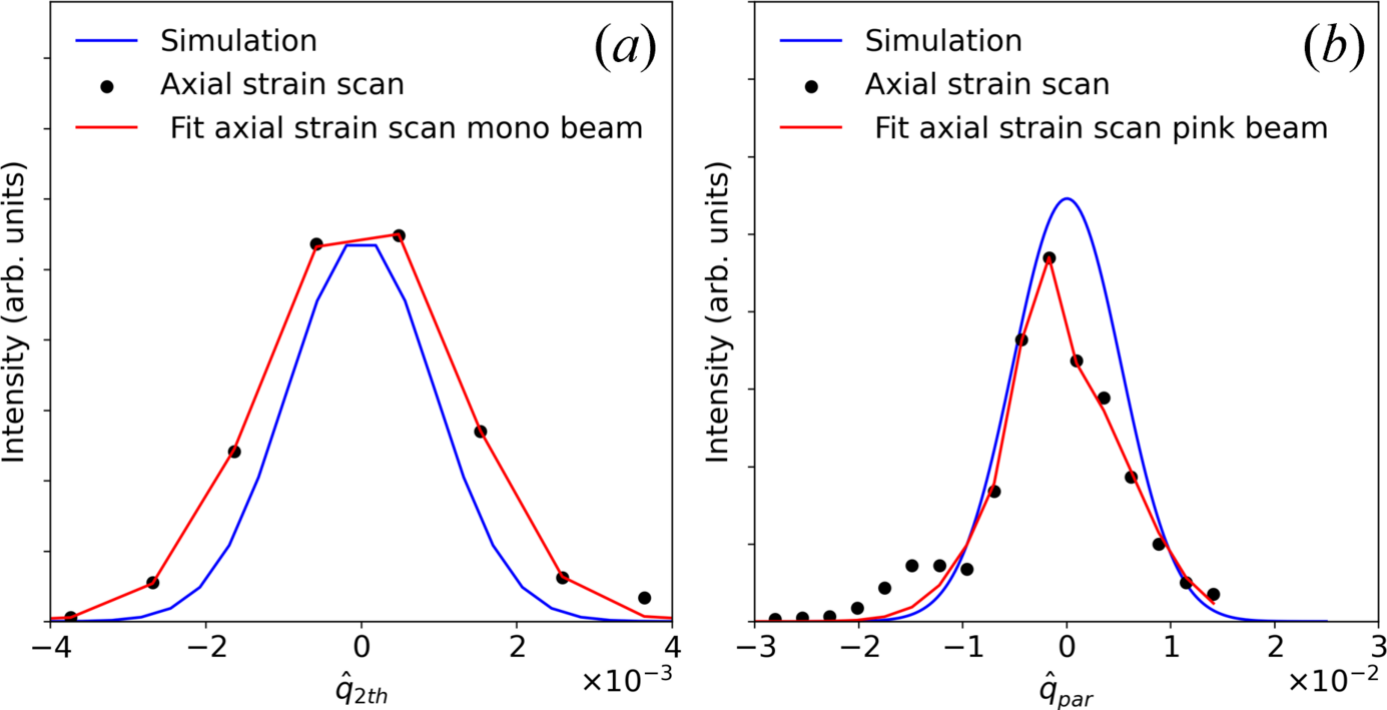
Comparison of normalized experimental data (dots) and simulations (blue curves) of the axial strain resolution function for the box beam case. Also shown are fits (red curves) of the experimental data. (*a*) Resolution function in 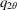 for a monochromatic beam. (*b*) Resolution function in 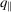 for a pink beam.

Fig. 6 ▸ shows corresponding data for the axial strain direction, probed by scanning the Bragg angle 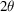 via a coupled motion of the objective and detector—which shifts the sampled position along the diffraction vector and is thus sensitive to changes in lattice spacing. Within the statistical uncertainty, a Gaussian model (blue curve) provides a reasonable approximation of the monochromatic beam data [Fig. 6 ▸(*a*)]. The pink-beam curve, however, deviates from a Gaussian shape due to the asymmetric energy profile of the pink beam (see SI Fig. S1), which is not captured by the symmetric Gaussian assumed in the simulation. The fitted FWHM values from both experimental and simulated datasets are listed in Table 1 ▸. The results overall confirm the expected reduction in resolution for the pink beam.

### 5.2. Chromatic aberration

The simulations in Section 4.2 indicate that the extent of blurring in direct space depends strongly on both the sample type and its local deformation state. At present, however, there is no established reference sample for DFXM comparable to the resolution target used in bright-field imaging. Consequently, a direct one-to-one validation of the theoretical predictions is not possible. Thus, to qualitatively evaluate the effects of chromatic aberration, we conducted two representative experiments using quasi-parallel (box beam) and condensed (line beam) illumination. The local spatial resolution was quantified using a Fourier-transform-based analysis, described in detail in SI Section 2, along with the experimental configurations.

**Figure 7:**
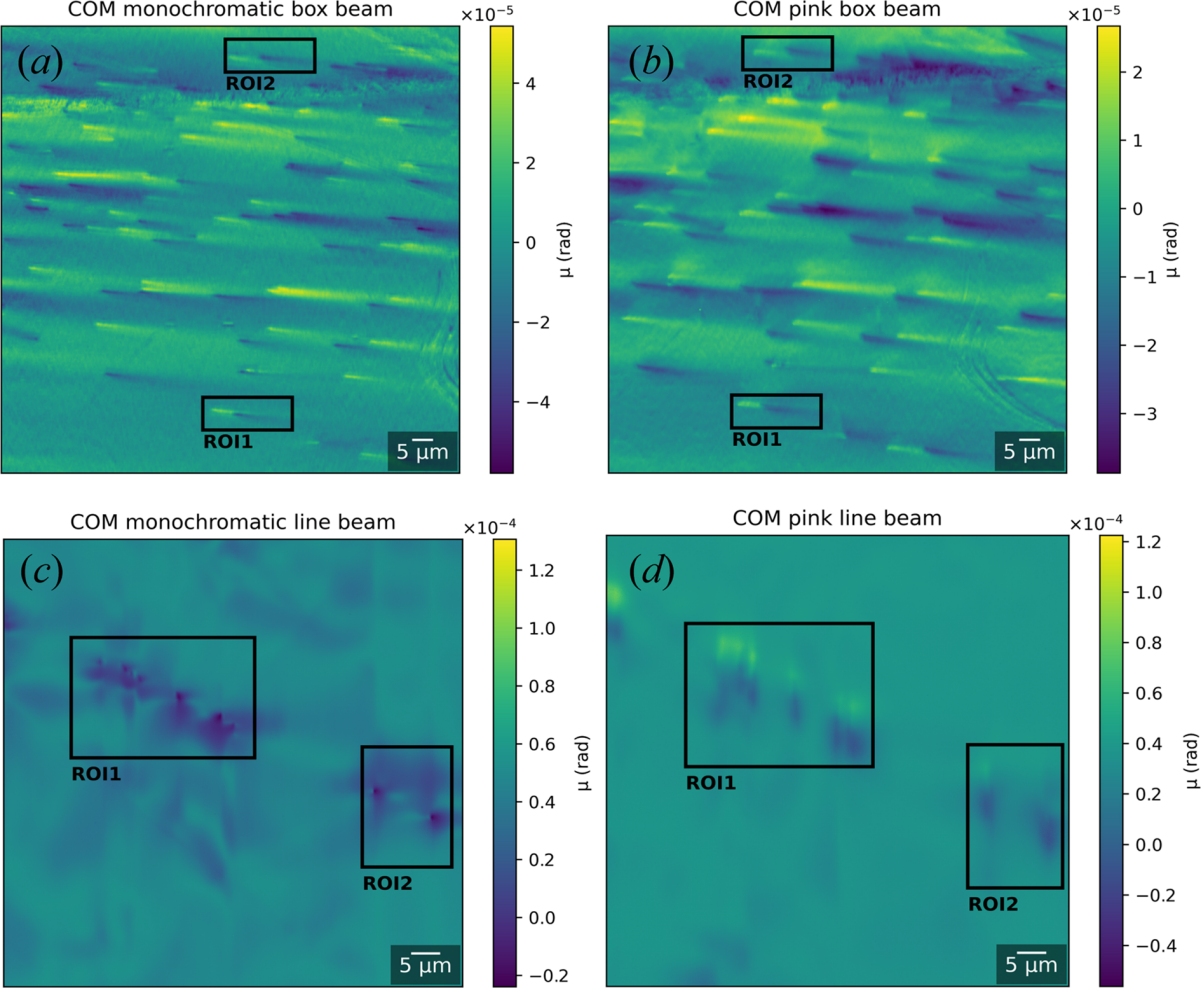
COM maps of the rocking curve measurements performed on (*a*) the BiFeO_3_ sample with a monochromatic box beam, (*b*) the BiFeO_3_ sample with a pink box beam, (*c*) the Si wafer sample with a monochromatic line beam and (*d*) the Si wafer sample with a pink line beam. The ROIs used for the quantification of the resolution are indicated with black rectangles.

*Box beam mode*. We produced center-of-mass (COM) maps from rocking scans of the thin-foil BiFeO_3_ sample using the *Darfix* software (Ferrer *et al.*, 2023 ▸). The data exhibited distinct and well-defined dislocations. We used these features to quantify the spatial resolution. Two regions of interest (ROIs), both containing single dislocations, were selected from the COM map. The resulting images, shown in Figs. 7 ▸(*a*) and 7 ▸(*b*), for monochromatic and pink beams, respectively, exhibited a lateral shift. Comparing the spatial resolutions obtained, we have found that the broader bandwidth of the pink-beam radiation only introduces a slight blur in the direct space image, leading to a decrease of resolution on the order of a few hundred nanometres. The values of resolution together with their standard deviations for the ROIs are listed in Table 3 ▸.

*Line beam mode*. In this case, we used a bulk Si wafer sample containing well-defined dislocations generated by indentation and annealing treatments. Again, we integrated the voxel intensities in a rocking scan. We focused on two ROIs at the center of the sample, presenting a network of well-known and previously studied dislocations (Kabukcuoglu, 2022 ▸). The COM maps shown in Figs. 7 ▸(*c*) and 7 ▸(*d*) were treated using the same protocol as for the box beam case. In this case, the decrease in resolution going from the monochromatic to the pink beam is on the order of 1–2 µm, higher than that of the box beam case. The results and standard deviations of the resolution quantification for both ROIs are listed in Table 3 ▸.

### 5.3. Weak-beam contrast

Mapping crystalline defects such as dislocations with DFXM has relied on the weak-beam contrast (Jakobsen *et al.*, 2019 ▸; Dresselhaus-Marais *et al.*, 2021 ▸; Yildirim *et al.*, 2023 ▸) enabled by the superior angular resolution in the rocking direction. Borgi *et al.* (2024 ▸) simulated the effect of increasing bandwidth on the weak-beam contrast. They generated images of a wall of edge dislocations with bandwidths increasing from 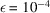 to 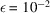, using a fixed incident divergence, corresponding to the use of the condenser. They also discussed the contrast as function of ϕ offset and proposed an 80% integrated intensity reduction as optimal.

**Figure 8:**
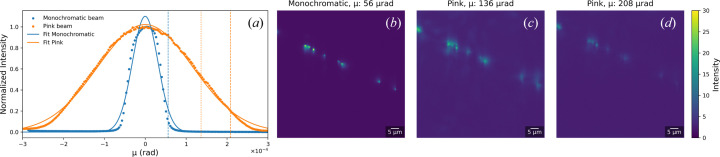
Weak-beam contrast for studying dislocations with line beam illumination in monochromatic and pink radiation cases. In this case the pink beam was attenuated by a 50 µm Cu foil. (*a*) Rocking curves obtained by summing intensities in each detector pixel over all images in the DFXM rocking scan. The intensity of the rocking curves was normalized to 1 to facilitate direct comparison. The blue curve was acquired with the monochromatic beam and the orange curve with the pink beam. Weak-beam contrast images: (*b*) Monochromatic beam at a rocking angle corresponding to 20% of the maximum intensity [blue dashed line in (*a*)]. (*c*) Pink beam at a rocking angle corresponding to HWHM [innermost orange dashed line in (*a*)]. (*d*) Pink beam at a rocking angle corresponding to 20% of the maximum intensity [outermost orange dashed line in (*a*)].

In an attempt to establish comparable experimental data we studied a chain of dislocations in the Si wafer sample, using the same datasets as presented above in connection with the direct space resolution quantification. The results for the line beam case are shown in Fig. 8 ▸. As discussed above, the rocking curve from the pink-beam illumination is in this case substantially wider than that from the monochromatic beam.

Comparing the results in Figs. 8 ▸(*b*), 8 ▸(*c*) and 8 ▸(*d*) we conclude that the ‘80%’ rule does not apply to pink beams. It is possible to acquire images without any (visible) effects of the strong beam at rocking angles corresponding to the HWHM of the rocking curve. This in conjunction with the high intensity in the pink beam implies that an improved S/N can be obtained in pink-beam mode, at the expense of a deterioration in spatial resolution. Corresponding results for the box beam case are provided in SI Section 3. While the 50% threshold identified here for the pink-beam case may not be universal across all samples, it provides a consistent and practical criterion for the Si dislocation dataset presented.

## 6. Effects of beam heating

Imaging at fourth-generation synchrotrons is known to cause sample heating. Regardless of S/N, pink-beam operation intensifies heating, as a larger fraction of the direct beam is not diffracted by the sample and instead contributes to absorption. The extent and structural impact of heating depends on the specimen and setup: in metals, dislocations become mobile before grain boundaries (Mavrikakis *et al.*, 2019 ▸; Yildirim *et al.*, 2022 ▸) (recovery precedes recrystallization and growth), while systems near phase transitions (Bucsek *et al.*, 2019 ▸) are especially sensitive. DFXM scans are also non-isothermal, as the beam shutter cycles during acquisition, causing transient thermal stresses that shift diffraction angles. Therefore, measurement results will depend not only on the overall length of the scans but also on the length of the waiting periods between consecutive exposures of the sample to the beam, caused by motor movements or data transfer, for example.

**Figure 9:**
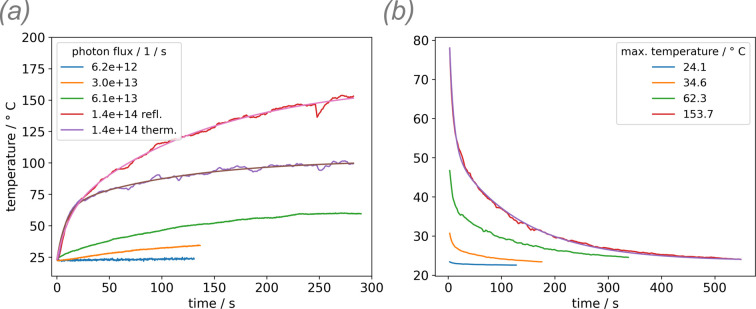
Temperature evolution during beam heating (*a*) and subsequent cooling (*b*) for an aluminium sample measured with a 17 keV pink beam. The purple heating curve and all cooling curves are measured by a thermocouple, while the other heating curves are measured by determining the shifts in the diffraction angle. The curves related to the highest photon flux are fitted to double-exponential fit functions.

To better understand such effects, we have focused on a mm-sized metal sample. For this, the characteristic internal diffusion time may be of order 1 ms, while the external heat transfer—*e.g.* to air—may be on the order of seconds or minutes. In the work of Bright *et al.* (2021 ▸), heating and passive cooling at the ID11 beamline of the ESRF were described in terms of a lumped thermodynamic model. We employed a similar approach to DFXM data at the ID03 beamline. Specifically, we investigated the effect of pink-beam exposure on the temperature of a 1.1 mm × 0.5 mm × 0.5 mm sized aluminium sample. The sample was illuminated with a box beam characterized by an area of approximately 0.19 mm^2^ at 17 keV. The incident beam had a photon flux of 1.615 × 10^14^ photons s^−1^ (assuming 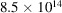
 photons s^−1^ for 1 mm × 1 mm slits). The cooling process was completely passive; no artificial cooling of the sample was employed. For this reason, sample cooling was due only to the heat exchange with the surrounding air at room temperature, the heat transfer inside the sample and thermal radiation. The sample was mounted on a metallic sample mount via ceramic glue. The interface between the sample and the mount was limited, but its contribution to the cooling process is most probably not negligible. During heating and cooling processes, the temperature was measured with a thermocouple attached to the sample in a region not exposed to the beam. The temperature of the illuminated subvolume was also calculated by tracking the 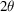 angle of the 111 reflection of one grain in the far-field detector. This permitted an accurate measurement of the heating temperature. Measurements were repeated by varying the exposure time between 0.01, 0.05, 0.1 and 0.5 s. The beamshutter was closed in between frame acquisitions for a certain amount of time, so the effective photon flux varied with the exposure time. The mean shutter closing times for the different exposure times were 0.25, 0.22, 0.17 and 0.07 s, respectively. The effective photon flux was calculated by multiplying the photon flux on the sample of 1.615 × 10^14^ photons s^−1^ by the ratio of the exposure time and shutter closing times. Results are shown in Fig. 9 ▸. The discrepancy during heating between the time evolution of the thermometer (purple curve) and the probe of the average temperature in the illuminated part of the sample (red curve) indicates that several processes with different time constants were at play, such as heterogeneous heat transfer inside the sample and transfer to the surrounding air. The curves can be well approximated by a double exponential function of the form
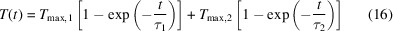
for both heating (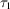) and cooling (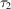). The lumped model predicts exponential temperature rise and decay over a single timescale (τ). For the curves in Fig. 9 ▸, this model breaks down and two timescales (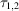) must be introduced to fit the data. Moreover, at around 250 s a local minimum in temperature can be seen for the highest-flux curve. Here, the time between two frames was about 2 s instead of the usual 0.5 s. This leads to a cooling of about 10 °C. The same result is apparent from the initial cooling in Fig 9 ▸(*b*).

Since only a part of the sample was illuminated [unlike in the work of Bright *et al.* (2021 ▸)] and the heat was transferred inside the sample, several different cooling mechanisms with different timescales contributed. As a result, the lumped model approach is not optimal for this system.

These results showcase three challenges for pink-beam operation in metals:

(1) The steady-state temperature in the unattenuated pink beam at 17 keV—using prefocusing by means of a transfocator but no condenser—is several hundred degrees and may lead to sample melting.

(2) The time to reach equilibrium may be 5–10 min [the red curve in Fig 9 ▸(*a*) has not yet saturated].

(3) The cooling time is sufficiently rapid (on the order of ∼10 °C s^−1^) that, for example, extended motor movements between single scans (with the shutter closed) can lead to a significant temperature drop and potential changes in microstructure and stress state. Any potential damage due to beam heating can be controlled by attenuating the incoming beam or by cooling the sample using constant gas/air flow.

## 7. Discussion

In the following, we discuss the implications of our findings, following the structure of the main body: reciprocal space resolution, chromatic aberration, weak-beam contrast and beam heating.

The degradation of angular resolution in the rocking and axial strain (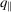) directions is a generic consequence of the broadened energy bandwidth, independent of sample type [equations (8) and (10), Tables 1 ▸ and 2 ▸]. For applications requiring high-resolution strain mapping, this represents a fundamental limitation. However, as demonstrated by Yildirim *et al.* (2025 ▸), the broader bandwidth can be advantageous: it enables integration over the full strain profile in a single exposure, accelerating experiments where integrated intensity rather than strain resolution is the priority. The effect of condensed versus box beam illumination on the reciprocal space resolution is negligible in the pink-beam case, which facilitates direct quantitative comparisons between the two illumination modes.

We foresee that for many science cases it is of relevance to swap between the use of a pink beam (overview scans over large orientation ranges and/or large sample volumes, fast acquisition during time series) and the use of a monochromatic beam (high spatial and angular resolution in general, strain scanning). At the ID03 beamline, switching between the two illumination schemes only takes a few minutes (Yildirim *et al.*, 2025 ▸).

An additional advantage of pink-beam operation may be to alleviate problems with dynamical diffraction—similar to work in electron backscatter diffraction (Winkelmann & Nolze, 2010 ▸) and pulsed electron microscopy. This is potentially an important argument for the use of pDFXM but is outside the scope of this work.

Regarding chromatic aberration, we find DFXM to be very different from bright-field microscopy. The fact that the sample effectively acts as a monochromator implies that for a perfect crystal, which will never be the case in reality, and for a near-parallel incident beam there is no aberration. This is of interest for magnified topography studies, *e.g.* inspection of rare defects in large single crystals. The introduction of a condenser, which is indispensable for many DFXM studies, leads to an anisotropic broadening, which still can be modeled for perfect crystals. For imperfect crystals, in particular plastically deformed ones, it will however be difficult to include the effects of aberration in forward modeling as it is not only strongly sample dependent but also dependent on the degree of mosaic spread within the individual voxel. For operation we propose to compare pDFXM and normal DFXM images directly to learn if the aberration is of concern.

In conventional monochromatic DFXM, the ϕ motor—or alternatively a base tilt μ—is used to map dislocations because of the high resolution in the rocking direction that provides good weak-beam conditions. In the case of the pink line beam illumination, one needs to increase the range of the rocking scan to reach the same intensity contrast as the monochromatic case. Nevertheless, as demonstrated in Fig. 8 ▸, the use of a pink beam ensures enough contrast in the weak-beam region for a clear detection of dislocations.

Imaging at fourth-generation synchrotrons causes significant sample heating, which is intensified in pink-beam operation. Our measurements on an aluminium sample (Fig. 9 ▸) show that the steady-state temperature can reach several hundred degrees, that equilibration times are on the order of 5–10 min and that cooling during shutter-closed intervals ∼10 °C s^−1^) can introduce transient thermal stresses that shift diffraction angles. As a result, measurements can depend on the total scan duration, motor speeds between frames and idle time with the shutter closed. Monitoring the sample temperature via a thermocouple or infrared camera, as well as tracking angular shifts of a Bragg reflection during a preliminary rocking scan, are practical strategies for characterizing and mitigating these effects.

Throughout this paper, we have assumed the use of CRL-based optics. Multilayer Laue lenses, MLLs, are an interesting alternative, due to the significantly larger NA, up to 0.016 for 20 keV operation (Braun *et al.*, 2013 ▸; Bajt *et al.*, 2017 ▸; Chapman *et al.*, 2021 ▸). At the same time, manufacturing errors can be reduced, implying that spatial resolution can be greatly improved in relation to CRL operation. For classical tomography, in bright-field mode, focal spots as small as 2.9 × 2.8 nm^2^ have been demonstrated at a photon energy of 17.5 keV (Dresselhaus *et al.*, 2024 ▸). Two crosslinked MLLs may be used as an objective for DFXM (Murray *et al.*, 2019 ▸; Kutsal *et al.*, 2019 ▸). The specifics of both monochromatic- and pink-beam properties of a DFXM instrument with such an objective are highly interesting but have yet to be derived theoretically. However, a basic understanding can be obtained from the equations derived here from the CRL case, simply by inserting the larger NA of the MLL. As an example, for the reciprocal space resolution function the 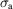 terms become dominant in the monochromatic-beam version of equations (8), (9) and (10), while the pink beam will be characterized by the fact that nearly the entire energy spread can be transmitted through the lens.

## 8. Conclusion

This work provides a rigorous framework for understanding and optimizing pDFXM. The central result is that the trade-offs introduced by the broader energy bandwidth are not uniform. They depend strongly on the illumination geometry, the sample perfection and the scientific question being asked. This makes informed experimental design essential for pDFXM users.

The tenfold degradation in angular resolution along the rocking and axial strain directions is an unavoidable consequence of the ∼100× broader bandwidth, ruling out high-resolution strain mapping but enabling efficient integration over broad orientation spreads in a single exposure. This is precisely the regime where monochromatic DFXM struggles most: heavily deformed grains, weakly diffracting crystals and time-resolved studies where speed matters more than strain sensitivity.

Perhaps the most surprising finding concerns chromatic aberration. Unlike bright-field X-ray microscopy, where aberration is a property of the optics alone, in dark-field mode the sample itself acts as a monochromator. For a perfect crystal under parallel (box beam) illumination, chromatic aberration is absent, even with a 1% energy bandwidth. Aberration only becomes significant when both a broad bandwidth and angular spread are present in the diffracted beam, either from the use of a condenser or from mosaic spread in deformed crystals. This has immediate practical consequences: box beam pDFXM of weakly deformed samples is essentially aberration free, while line beam operation on plastically deformed crystals introduces spatially varying blur that complicates forward modeling. For the latter case, direct comparison with monochromatic images remains the most reliable diagnostic.

Weak-beam imaging of dislocations remains feasible under pink-beam operation, with the HWHM of the rocking curve providing adequate contrast; the ‘80%’ rule from monochromatic DFXM does not apply. The higher flux can yield improved signal-to-noise ratios for dislocation detection, at the cost of spatial resolution.

Beam heating is a practical concern that must not be overlooked. At 17 keV, unattenuated pink-beam illumination can raise the temperature of metallic samples by several hundred degrees, with equilibration times of 5–10 min and fast cooling transients during shutter-closed intervals. However, this challenge is not insurmountable. Attenuation preserves a significant flux advantage over monochromatic operation, and the use of box beam illumination, which avoids the condenser and distributes the thermal load over a larger area, naturally mitigates both heating and chromatic aberration simultaneously. Most promisingly, increasing the photon energy dramatically reduces absorption and hence heating. The ongoing transition from beryllium to diamond compound refractive lenses (Staeck *et al.*, 2026 ▸) will support routine operation at higher energies (35–40 keV), opening the door to pDFXM studies of thicker, denser and more radiation-sensitive materials.

Looking ahead, the combination of pink beams with magnified topotomography (Shukla *et al.*, 2025 ▸) is particularly exciting. Box beam pDFXM is inherently compatible with tomographic acquisition, and the high flux enables sub-second frame rates even at reduced ring currents. This makes true 4D imaging, 3D mapping with time resolution on the scale of seconds, a realistic prospect for studying grain growth, recrystallization kinetics and phase transformations *in situ*. Beyond synchrotron sources, the formalism developed here applies equally to X-ray free-electron lasers (XFELs), where the self-amplified spontaneous emission process produces intrinsically polychromatic pulses with 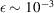, intermediate between the monochromatic- and pink-beam cases. The femtosecond pulse duration of XFELs could ultimately enable single-shot pDFXM, pushing time-resolved diffraction imaging into entirely new regimes.

## Supplementary Material

Supplementary information. DOI: 10.1107/S1600576726005200/man5003sup1.pdf

## Figures and Tables

**Table 1:** Widths (FWHM) of the experimental and simulated strain resolution function in the box beam case

Radiation	Experimental data	FWHM	Simulation	FWHM
Mono	Rocking	0.00003	Rocking	0.00003
Mono	Rolling	0.00145	Rolling	0.00202
Mono	Axial strain	0.00288	Axial strain	0.00222

Pink	Rocking	0.00031	Rocking	0.00035
Pink	Rolling	0.00173	Rolling	0.00199
Pink	Axial strain	0.01026	Axial strain	0.01227

**Table 2:** Widths (FWHM) of the experimental and simulated strain resolution function in the line beam case

Radiation	Experimental data	FWHM	Simulation	FWHM
Mono	Rocking	0.00026	Rocking	0.00023
Mono	Rolling	0.00174	Rolling	0.00201

Pink	Rocking	0.00032	Rocking	0.00037
Pink	Rolling	0.00180	Rolling	0.00200

**Table 3:** Quantified dark-field direct space resolution, with its standard deviation, in the box and line beam illumination modes

Illumination	Radiation	ROI	Resolution (nm)
Box beam	Monochromatic	1	529 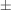 19
		2	821 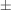 58
	Pink	1	799 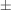 80
		2	959 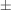 81
Line beam	Monochromatic	1	1984 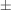 284
		2	1600 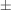 443
	Pink	1	3266 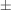 149
		2	4127 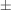 106

## References

[bb1] Ahl, S., Simons, H., Zhang, Y., Detlefs, C., Stöhr, F., Jakobsen, A., Juul Jensen, D. & Poulsen, H. (2017). *Scr. Mater.* **139**, 87–91.

[bb2] Bajt, S., Prasciolu, M., Fleckenstein, H., Domaracký, M., Chapman, H. N., Morgan, A. J., Yefanov, O., Messerschmidt, M., Du, Y., Murray, K. T., Mariani, V., Kuhn, M., Aplin, S., Pande, K., Villanueva-Perez, P., Stachnik, K., Chen, J. P., Andrejczuk, A., Meents, A., Burkhardt, A., Pennicard, D., Huang, X., Yan, H., Nazaretski, E., Chu, Y. S. & Hamm, C. E. (2017). *Light Sci. Appl.* **7**, 17162. 10.1038/lsa.2017.162 PMC6060042 30839543

[bb3] Borgi, S., Ræder, T. M., Carlsen, M. A., Detlefs, C., Winther, G. & Poulsen, H. F. (2024). *J. Appl. Cryst.* **57**, 358–368. 10.1107/S1600576724001183 PMC11001414 38596724

[bb4] Braun, S., Kubec, A., Menzel, M., Niese, S., Krüger, P., Seiboth, F., Patommel, J. & Schroer, C. (2013). *J. Phys. Conf. Ser.* **425**, 052019.

[bb6] Bucsek, A., Seiner, H., Simons, H., Yildirim, C., Cook, P., Chumlyakov, Y., Detlefs, C. & Stebner, A. P. (2019). *Acta Mater.* **179**, 273–286.

[bb7] Chapman, H. N., Prasciolu, M., Murray, K. T., Lukas Dresselhaus, J. & Bajt, S. (2021). *Opt. Express* **29**, 3097–3113. 10.1364/OE.413916 33770916

[bb8] Chen, Y., Tang, Y. T., Collins, D. M., Clark, S. J., Ludwig, W., Rodriguez-Lamas, R., Detlefs, C., Reed, R. C., Lee, P. D., Withers, P. J. & Yildirim, C. (2023). *Scr. Mater.* **234**, 115579.

[bb42] Chu, Y., Cruz, M., Yang, C., Martin, L., Yang, P., Zhang, K., Lee, P., Yu, P., Chen, L. & Ramesh, R. (2007). *Adv. Mater.* **19**, 2662–2666.

[bb9] Cook, P. K., Simons, H., Jakobsen, A. C., Yildirim, C., Poulsen, H. F. & Detlefs, C. (2018). *Microsc. Microanal.* **24**(S2), 88–89.

[bb10] Detlefs, C., Henningsson, A., Kanesalingam, B., Cretton, A. A. W., Corley-Wiciak, C., Frankus, F. T., Pal, D., Irvine, S., Borgi, S., Poulsen, H. F., Yildirim, C. & Dresselhaus-Marais, L. E. (2025). *J. Appl. Cryst.* **58**, 1439–1446. 10.1107/S1600576725005862 PMC12321021 40765970

[bb11] Dresselhaus, J. L., Zakharova, M., Ivanov, N., Fleckenstein, H., Prasciolu, M., Yefanov, O., Li, C., Zhang, W., Middendorf, P., Egorov, D., De Gennaro Aquino, I., Chapman, H. N. & Bajt, S. (2024). *Opt. Express* **32**, 16004–16015. 10.1364/OE.518964 38859238

[bb12] Dresselhaus-Marais, L. E., Winther, G., Howard, M., Gonzalez, A., Breckling, S. R., Yildirim, C., Cook, P. K., Kutsal, M., Simons, H., Detlefs, C., Eggert, J. H. & Poulsen, H. F. (2021). *Sci. Adv.* **7**, eabe8311. 10.1126/sciadv.abe8311 PMC8279502 34261647

[bb13] Falch, K., Detlefs, C., Di Michiel, M., Snigireva, I., Snigirev, A. & Mathiesen, R. (2016). *Appl. Phys. Lett.* **109**, 054103.

[bb14] Garriga Ferrer, J., Rodríguez-Lamas, R., Payno, H., De Nolf, W., Cook, P., Solé Jover, V. A., Yildirim, C. & Detlefs, C. (2023). *J. Synchrotron Rad.* **30**, 527–537. 10.1107/S1600577523001674 PMC10161887 37000183

[bb15] Gürsoy, D., Yay, K. A., Kisiel, E., Wojcik, M., Sheyfer, D., Last, A., Highland, M., Fisher, I. R., Hruszkewycz, S. & Islam, Z. (2025). *Commun. Phys.* **8**, 34.

[bb16] Gustafson, S., Ludwig, W., Shade, P., Naragani, D., Pagan, D., Cook, P., Yildirim, C., Detlefs, C. & Sangid, M. D. (2020). *Nat. Commun.* **11**, 3189. 10.1038/s41467-020-16894-2 PMC7314802 32581264

[bb17] Gustafson, S. E., Ludwig, W., Rodriguez-Lamas, R., Yildirim, C., Shanks, K. S., Detlefs, C. & Sangid, M. D. (2023). *Acta Mater.* **260**, 119300.

[bb18] Henningsson, A., Borgi, S., Winther, G., El-Azab, A. & Poulsen, H. F. (2025). *J. Mech. Phys. Solids* **204**, 106277.

[bb19] Holstad, T. S., Dresselhaus-Marais, L. E., Raeder, T. M., Kozioziemski, B., Driel, T. V., Seaberg, M., Folsom, E., Eggert, J. H., Knudsen, E. B., Nielsen, M. M., Simons, H., Haldrup, K. & Poulsen, H. F. (2023). *Proc. Natl Acad. Sci. USA* **120**, e2307049120. 10.1073/pnas.2307049120 PMC10523471 37725646

[bb20] Isern, H., Brochard, T., Dufrane, T., Brumund, P., Papillon, E., Scortani, D., Hino, R., Yildirim, C., Rodriguez Lamas, R., Li, Y., Sarkis, M. & Detlefs, C. (2025). *J. Phys. Conf. Ser.* **3010**, 012163.

[bb21] Jakobsen, A. C., Simons, H., Ludwig, W., Yildirim, C., Leemreize, H., Porz, L., Detlefs, C. & Poulsen, H. F. (2019). *J. Appl. Cryst.* **52**, 122–132.

[bb22] Kabukcuoglu, M. P. (2022). PhD thesis, Universität Freiburg, Germany.

[bb23] Kutsal, M., Bernard, P., Berruyer, G., Cook, P. K., Hino, R., Jakobsen, A. C., Ludwig, W., Ormstrup, J., Roth, T., Simons, H., Smets, K., Sierra, J. X., Wade, J., Wattecamps, P., Yildirim, C., Poulsen, H. F. & Detlefs, C. (2019). *IOP Conf. Ser. Mater. Sci. Eng.* **580**, 012007.

[bb5] Lawrence Bright, E., Giacobbe, C. & Wright, J. P. (2021). *J. Synchrotron Rad.* **28**, 1377–1385. 10.1107/S160057752100669X PMC8415326 34475286

[bb24] Lee, S., Berman, T. D., Yildirim, C., Detlefs, C., Allison, J. E. & Bucsek, A. (2024). *Sci. Rep.* **14**, 6241. 10.1038/s41598-024-56546-9 PMC10940669 38486085

[bb25] Lee, S., Pilipchuk, M., Yildirim, C., Greeley, D., Shi, Q., Berman, T., Creuziger, A., Rust, E., Detlefs, C., Sundararaghavan, V., Allison, J. E. & Bucsek, A. (2025). *Science*. In the press. 10.1126/science.adv3460 40773550

[bb26] Mavrikakis, N., Detlefs, C., Cook, P., Kutsal, M., Campos, A., Gauvin, M., Calvillo, P., Saikaly, W., Hubert, R., Poulsen, H., Vaugeois, A., Zapolsky, H., Mangelinck, D., Dumont, M. & Yildirim, C. (2019). *Acta Mater.* **174**, 92–104.

[bb27] Murray, K. T., Pedersen, A. F., Mohacsi, I., Detlefs, C., Morgan, A. J., Prasciolu, M., Yildirim, C., Simons, H., Jakobsen, A. C., Chapman, H. N., Poulsen, H. F. & Bajt, S. (2019). *Opt. Express* **27**, 7120–7138. 10.1364/OE.27.007120 30876283

[bb28] Poulsen, H. F., Cook, P. K., Leemreize, H., Pedersen, A. F., Yildirim, C., Kutsal, M., Jakobsen, A. C., Trujillo, J. X., Ormstrup, J. & Detlefs, C. (2018). *J. Appl. Cryst.* **51**, 1428–1436.

[bb29] Poulsen, H. F., Dresselhaus-Marais, L. E., Carlsen, M. A., Detlefs, C. & Winther, G. (2021). *J. Appl. Cryst.* **54**, 1555–1571.

[bb30] Poulsen, H. F., Jakobsen, A. C., Simons, H., Ahl, S. R., Cook, P. K. & Detlefs, C. (2017). *J. Appl. Cryst.* **50**, 1441–1456.

[bb31] Shukla, A., Yildirim, C., Ball, J. A. D., Detlefs, C., Cretton, A. A. W., Sarkis, M., Bella, M. L., Ludwig, W., Zhang, Y. & Henningsson, N. A. (2025). *arXiv*, 2508.17897.

[bb32] Simons, H., Ahl, S. R., Poulsen, H. F. & Detlefs, C. (2017). *J. Synchrotron Rad.* **24**, 392–401. 10.1107/S160057751602049X PMC5330290 28244432

[bb33] Simons, H., Haugen, A. B., Jakobsen, A. C., Schmidt, S., Stöhr, F., Majkut, M., Detlefs, C., Daniels, J. E., Damjanovic, D. & Poulsen, H. F. (2018). *Nat. Mater.* **17**, 814–819. 10.1038/s41563-018-0116-3 29941920

[bb41] Simons, H., Jakobsen, A. C., Ahl, S. R., Poulsen, H. F., Pantleon, W., Chu, Y. H., Detlefs, C. & Valanoor, N. (2019). *Nano Lett.* **19**, 1445–1450. 10.1021/acs.nanolett.8b03839 30724569

[bb34] Simons, H., King, A., Ludwig, W., Detlefs, C., Pantleon, W., Schmidt, S., Stöhr, F., Snigireva, I., Snigirev, A. & Poulsen, H. F. (2015). *Nat. Commun.* **6**, 6098. 10.1038/ncomms7098 PMC4354092 25586429

[bb35] Snigirev, A., Kohn, V. G., Snigireva, I. I. & Lengeler, B. (1996). *Nature* **384**, 49–51.

[bb51] Staeck, S., Yildirim, C., Seiboth, F., Manning, T., Roth, T., Stinville, J.-C. & Detlefs, C. (2026). *arXiv*, 2605.26251.

[bb36] Winkelmann, A. & Nolze, G. (2010). *Ultramicroscopy* **110**, 190–194. 10.1016/j.ultramic.2009.11.008 20005045

[bb50] Yildirim, C., Mavrikakis, N., Cook, P. K., Rodriguez-Lamas, R., Kutsal, M., Poulsen, H. F. & Detlefs, C. (2022). *Scr. Mater.* **214**, 114689.

[bb37] Yildirim, C., Poulsen, H. F., Winther, G., Detlefs, C., Huang, P. H. & Dresselhaus-Marais, L. E. (2023). *Sci. Rep.* **13**, 3834. 10.1038/s41598-023-30767-w PMC9992398 36882517

[bb38] Yildirim, C., Shukla, A., Zhang, Y., Mavrikakis, N., Lesage, L., Sanna, V., Sarkis, M., Li, Y., La Bella, M., Detlefs, C. & Poulsen, H. F. (2025). *arXiv*, 2503.05921. 10.1038/s43246-025-00926-9 PMC12396957 40895772

[bb39] Zelenika, A., Cretton, A. A. W., Frankus, F., Borgi, S., Grumsen, F. B., Yildirim, C., Detlefs, C., Winther, G. & Poulsen, H. F. (2025). *Sci. Rep.* **15**, 8655. 10.1038/s41598-025-88262-3 PMC11906882 40082456

[bb40] Zhou, T., Reinhardt, A., Bousquet, M., Eymery, J., Leake, S., Holt, M. V., Evans, P. G. & Schülli, T. (2025). *Nat. Commun.* **16**, 2822. 10.1038/s41467-025-57814-6 PMC11929839 40121198

