# Supplementary material for: Quantifying resolution in pink-beam dark-field X-ray microscopy: experiments and simulations
Source: J Appl Crystallogr. 2026 Jul 7;59(Pt 4):1139–52. doi: 10.1107/S1600576726005200 (PMC13440070; doi:10.1107/S1600576726005200)
Supplement: Supplementary file 1 [file j-59-01139-sup1.pdf]

JOURNAL OF APPLIED CRYSTALLOGRAPHY

Volume 59 (2026)

Supporting information for article:

Quantifying resolution in pink-beam dark-field X-ray microscopy: experiments and simulations

M. La Bella, H. F. Poulsen, S. Staeck, N. A. Henningsson, M. P. Kabukcuoglu, C. Detlefs and C. Yildirim

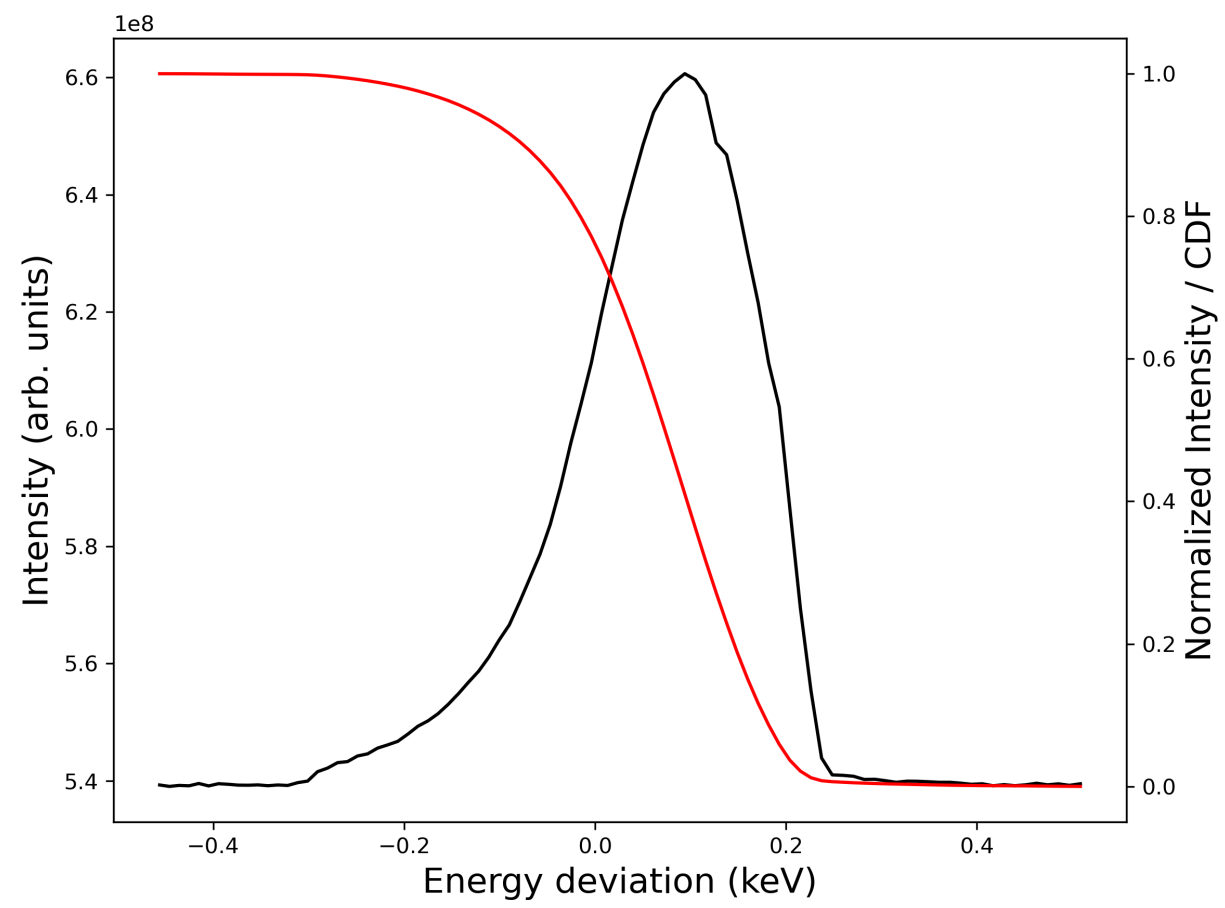

Figure S1: Energy profile of the pink beam entering the experimental hutch at ID03, ESRF, for an average energy of 17.2 keV. The black curve represents the integrated intensities extracted from the images recorded in the near field camera. The red curve is the corresponding Cumulative Distribution Function, CDF. The FWHM determined from the CDF is 0.208 keV.

1 Reciprocal space resolution

1.1 Pink beam energy profile

The energy profile associated with the multilayer monochromator (pink beam) was determined experimentally, at 17.2 keV, by a "rocking scan" of a reference sample consisting of a 120-nm thick layer of $BiFeO_3$ grown on a substrate. (Details about sample manufacturing can be found in [1] and [2]). The sample was oriented such that the (002) reflection was in the Laue conditions. Images were collected with a box beam in the near-field camera. The rocking curve was extracted by summing the intensity in all pixels in each recorded image. The motor positions were then converted into strain unit values and successively into energy to calculate the energy shift ΔE. The experimental curve is shown in Fig. S1. The FWHM was determined from the cumulative distribution function, resulting in a value of FWHM = 0.2 keV. Hence, $\epsilon = 1.2$ %. The value of energy spread obtained was used for all the simulations.

1.2 Rocking and rolling directions

A diamond sample was oriented on the instrument so that the (111) reflection was in Laue conditions, giving rise to a scattering angle of $2\theta = 20.4°$ Two sets of measurements were performed for both monochromatic and pink beam configurations in the box and line beam settings. For the monochromatic beam, the energy spread was set to $\sigma_e = 6 \times 10^{-5}$ r.m.s. following [3]. The divergence of the horizontal incident beam was set to $\xi_h = 0.01$ mrad, corresponding to the use of an upstream transfocator. The divergence of the vertical incident beam was $\xi_v = 0.53$ mrad and $\xi_v = 0.01$ mrad in the case of an incident line beam and a box

beam, respectively. The numerical aperture of the objective is $\sigma_a = 3.11 \times 10^{-4}$ rad in all cases. The CRL, equipped with 87 2D Be lenses with R = 50 μm and T = 1.6 mm. With $d_1 + d_2 = 5000$ m, the resulting magnification of the diffracted beam was $\mathcal{M} = -19.39$. In the case of the pink line beam configuration, two Cu foils with 50 μm thickness were used as attenuators. Rocking and rolling scans were acquired by moving the ϕ and χ motors, respectively.

For the comparison with the simulations, the experimental curves were extracted from the raw data by summing the intensity of the pixels in each image. The values of intensity were plotted, after background subtraction and fitted with Gaussian models to calculate the FWHM.

For completeness, we mention that the incident fluxes of the monochromatic and pink beams were measured by means of an ion chamber to be 6.6×10^{12} photons/s and 8.44×10^{14} photons/s, respectively. These values are in line with those reported in [4].

1.3 Axial strain direction

The experimental angular resolution in the axial strain direction was measured using the same $BiFeO_3$ sample, at an energy of 19.2 keV, which was also used for the beam profile measurements. In this case, the rocking curves were acquired by a combined motion of the CRL and the arm of the detector while scanning the μ motor at each step. Two measurements were done, one with the monochromatic beam and one with the pink beam. In both cases, the beam was in the box beam setting. For each position of the CRL-detector, a rocking curve in μ was acquired. In the case of the monochromatic beam, the experimental curve is drawn by 16 measurement points which makes it less smooth compared to the other curves in the rocking and rolling directiuons. In the case of the pink beam, the shape of the curve, made by 19 measurement points is not Gaussian. To obtain the curves that are fitted and shown in Fig.9, we first plotted all the μ rocking curves. The sum of all the μ rocking curves for monochromatic (a) and pink (b) radiations are reported in Fig.S2. To better understand how these data were acquired and treated, intensity maps relative to the same data are also plotted for the monochromatic and pink beam, respectively in Fig.S2c-d. Successively, we extracted the maximum value of each μ rocking curve, and we obtained the axial strain maps for both cases.

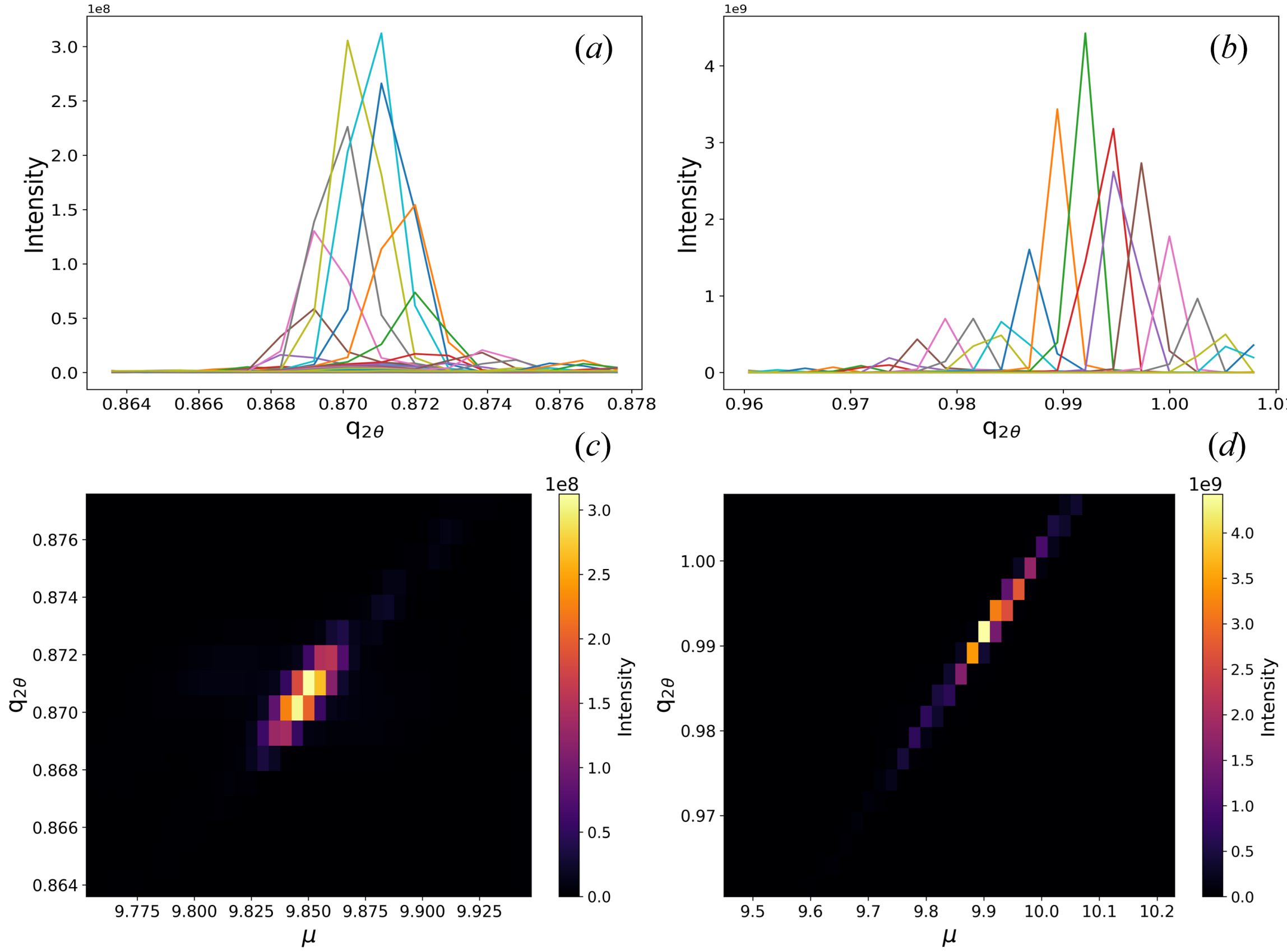

Figure S2: (a) Sum of the rocking curves in μ obtained for each step of the CRL-detector angular range with monochromatic beam. (b) same as (a) in the case of pink beam. (c) Intensity map of (a) showing both the ranges of the μ and CRL-detector ($q_{2\theta}$) motors. (d) same as (c) in the case of pink beam.

2 Direct space resolution in dark-field

The dataset used to quantify the resolution with the parallel beam illumination was acquired using the $BiFeO_3$ sample. The data were taken in reflection mode, diffracting on the (002) planes at an X-ray energy of 19.2 keV. The measurements were performed using consistent parameters, including an angular step of 1.7453 μrad, 400 steps, and an exposure time of 0.5 s. In the pink beam case, we have attenuated the incident radiation with a 50 μm Cu foil. The objective that we used to magnify the reflection was the 10x, resulting in a magnification of $\mathcal{M} = 11.26$ and an effective pixel size of 0.058 μm.
In the case of the condensed illumination mode, we used the Si wafer sample and we diffracted the (220) planes at an energy of 19.2 keV. The beam was shaped with the condenser also here, and the pink beam attenuated with the Cu foil. For the measurements with monochromatic and pink beams, we used an angular step of 3.49 μrad. We took 200 and 300 steps for the monochromatic and pink beam, respectively, and an exposure time of 1s. We used the

10x objective yielding a $\mathcal{M} = 15.77$ and an effective pixel size of 0.041 μm.
Both the datasets for the parallel (box) and condensed (line) illuminations acquired with monochromatic and pink radiations have been analyzed using the same procedure. Center-of-mass (COM) maps for all datasets were reconstructed using the software package Darfix [5]. The COM maps were successively detrended, **i.e. corrected for slowly varying intensity gradients imposed by the acquisition geometry**. To quantify the spatial resolution across the FOV, we have selected several ROIs, including single or groups of dislocations in different areas of the maps. These 2D areas were analysed using a Fourier space approach. We reconstructed the power spectrum of the ROIs. Then, in the Fourier space, we averaged the signal around an angular wedge of 20° in the vertical direction, and we extracted 1D directional spectra. The spectra were normalized, and we defined an intensity cutoff threshold to discard signals arising from noise. The frequency associated with the dislocations in the ROIs was then used to calculate the resolution in that specific area of the FOV. The resolution in pixels was calculated in this way:

$$r = \frac{1}{2f_c}. \tag{1}$$

With f_c being the value of frequency defined by the cutoff. The value in unit length was then retrieved using the effective pixel size of the measurements. The uncertainty in the resolution was estimated as the standard deviation of the resolution values obtained when varying the angular wedge between 5° and 55° in 5° steps, while keeping the orientation fixed along the y-direction. This standard deviation reflects the sensitivity of the resolution estimate to the choice of wedge width. Fig.S3 shows an example of the steps of the procedure.

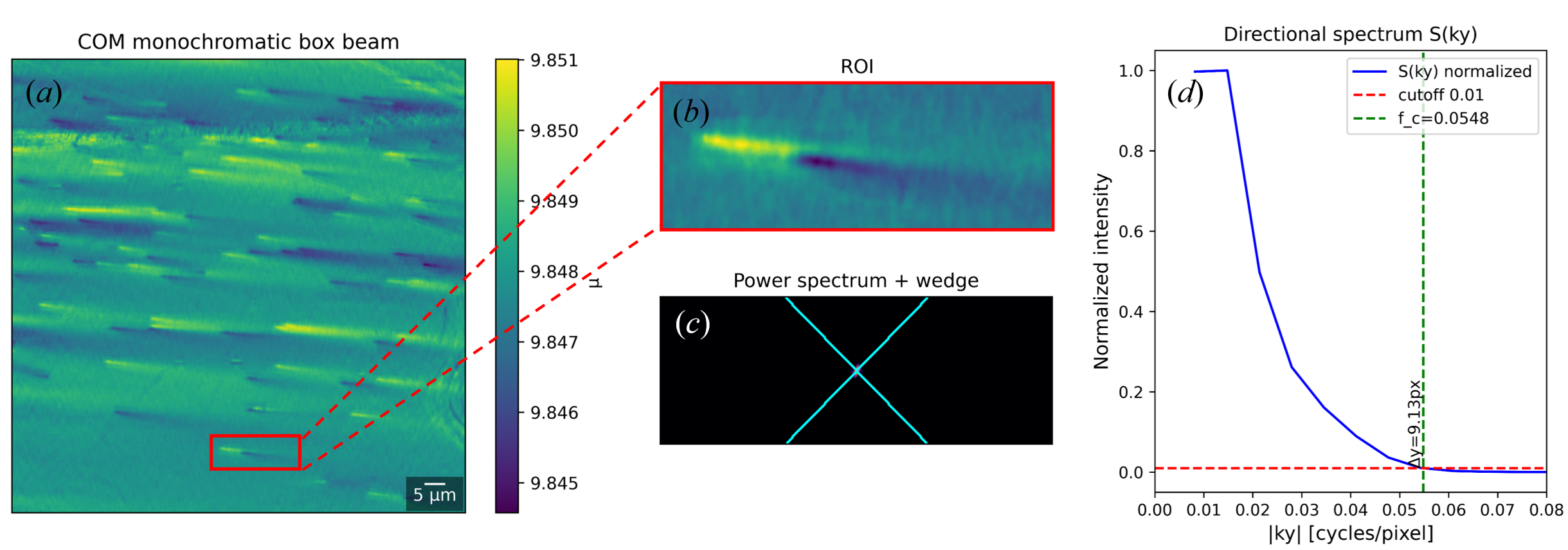

Figure S3: a) COM map from the rocking curve scan done with the monochromatic beam and parallel illumination. b) Zoom on a selected ROI containing a dislocation that was used to determine the resolution. c) Power spectrum of b with wedge of 20 degrees used to calculate the directional spectrum in the vertical (y) direction. d) Directional spectrum of the 2D area showing the f_c value corresponding to a resolution of 9.13 pixels obtained with a cutoff in intensity of 0.01.

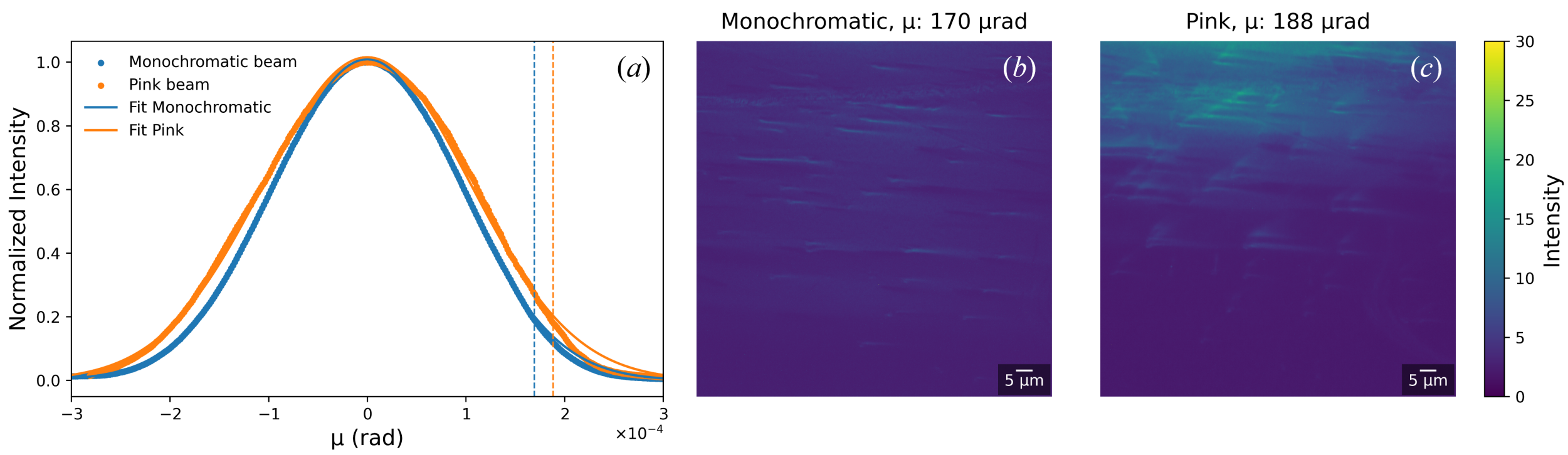

Figure S4: Weak beam contrast for box beam illumination in monochromatic and pink radiation cases. a) Rocking curves obtained by integrating over the μ motor. The intensity was normalized after being extracted from each image recorded in the far-field detector. The blue curve was acquired with the monochromatic beam, and the orange curve with the pink beam. b) Weak beam contrast image corresponding to the 20% of the intensity of the rocking curve in monochromatic illumination. c) Same as b with pink illumination.

3 Weak beam contrast with box beam

The weak beam contrast associated with the use of pink beam has also been investigated in the box beam case. To do so, we have used the same dataset obtained with the $BiFeO_3$ sample presented in the direct space resolution study. Fig.S4 shows the results. The two rocking curves obtained with monochromatic (blue) and pink (orange) radiations in Fig.S4a, after background subtraction and intensity normalization, were fitted to a Gaussian model to identify the motor position corresponding to 20% of the intensity. In the case of the monochromatic beam (Fig. S4b), the weak beam conditions are found at $\mu = 170$ μrad, while for the pink beam (Fig. S4c), at $\mu = 188$ μrad.

4 Direct space resolution in bright-field

The direct space resolution in bright field conditions ($2\theta = 0$) was investigated using a JIMA target. The target had known dimensions, with pillars of 400 nm width separated by a period of 200 nm. We slid the target across the FOV in the horizontal direction. A total of 51 images were acquired with a spacing of 2 μm to cover the full FOV. The measurements were done at an energy of 17 keV using a consistent exposure time of 0.1 s. With these datasets, two strategies were used to investigate the change in contrast between the two types of illuminations and the blur due to chromatic aberrations introduced by the pink beam.

Quantification of contrast across the FOV: We extracted horizontal profiles of the JIMA oscillations in each image recorded. The profiles were extracted at the center of the FOV, averaging over 100 pixels. We applied the FFT method to identify the frequency component associated with the JIMA target pillars. Fig.S5 shows the full workflow of the analyses.

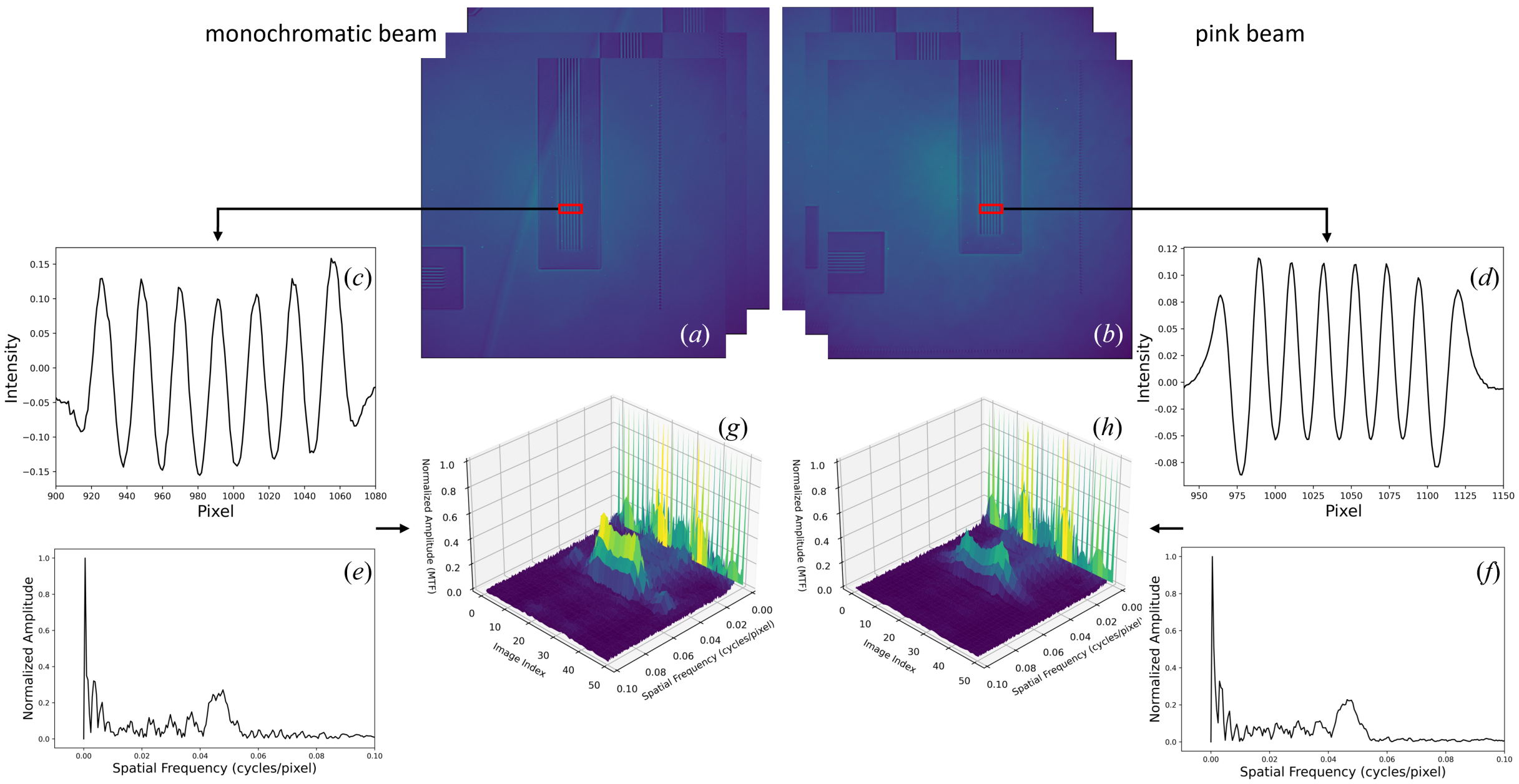

Figure S5: Schematic representation of the full procedure used for the FFT analysis. a-b) Sequence of images recorded in the far-field detector with the monochromatic and pink beam, respectively. c-d) Horizontal intensity profiles of the JIMA pillars obtained by averaging 10 pixels in the monochromatic and pink cases, respectively. e-f) FFT of the oscillations isolated in c-d. g-h) 3D plots of the FFT signals from all the images for the monochromatic and pink beam data, respectively.

Fig.S5a-b displays a few of the direct space images with the JIMA target that were acquired with both the monochromatic (a) and pink (b) beam. Fig.S5c-d shows cuts of the horizontal profiles with the oscillations of the JIMA target. These regions were Fourier transformed as shown in Fig.S5e-f. The full treated datasets are plotted in Fig.S5g-h. Already by qualitatively comparing the 3D plots, we can spot the differences between the monochromatic and pink beam cases. The peak in the FFT plots located around 0.04 cycles/pixel corresponds to the oscillations of the JIMA target. With the monochromatic beam, the amplitude of the peak corresponding to the image where the JIMA is at the center of the FOV is higher compared to the pink beam. This indicates that the contrast of the JIMA target features is higher in the case of the monochromatic beam. In both cases, the amplitude of the FFT decreases as the JIMA target moves away from the center. To quantify the visibility of the fringes relative to the JIMA target pillars, we calculated the Michelson contrast for each image. The Michelson contrast was calculated in this way:

$$C = \frac{I_{\max} - I_{\min}}{I_{\max} + I_{\min}} \tag{2}$$

Where $I_{\max}$, and $I_{\min}$ are the maximum and minimum intensity of the profiles. C should range between 0 and 1, which are the values related to a completely blurred image and the

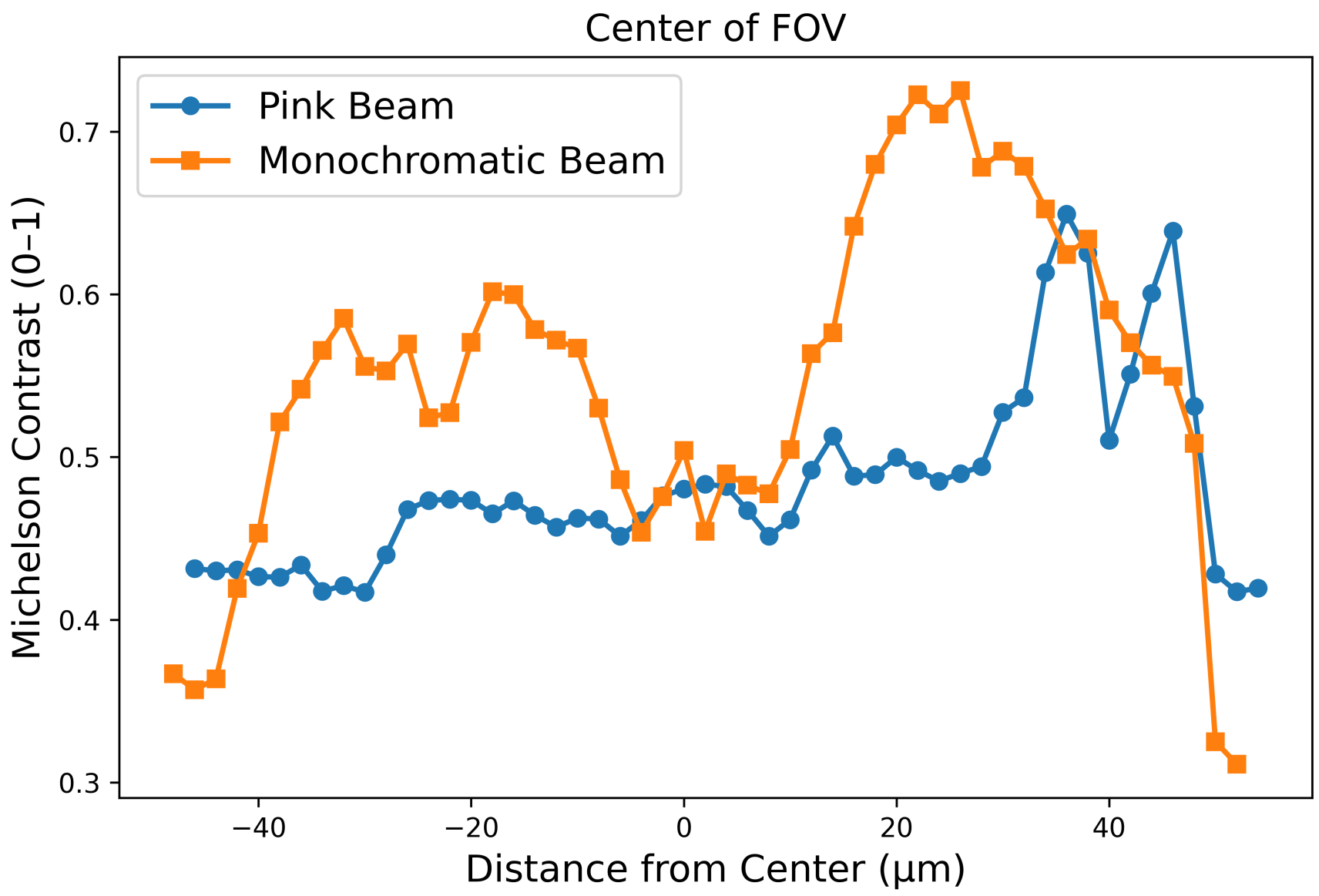

Figure S6: Michelson contrast as a function of the distance of the JIMA target from the center of the FOV. The contrast from the images acquired with the monochromatic beam is reported in orange, and the one with the pink beam in blue.

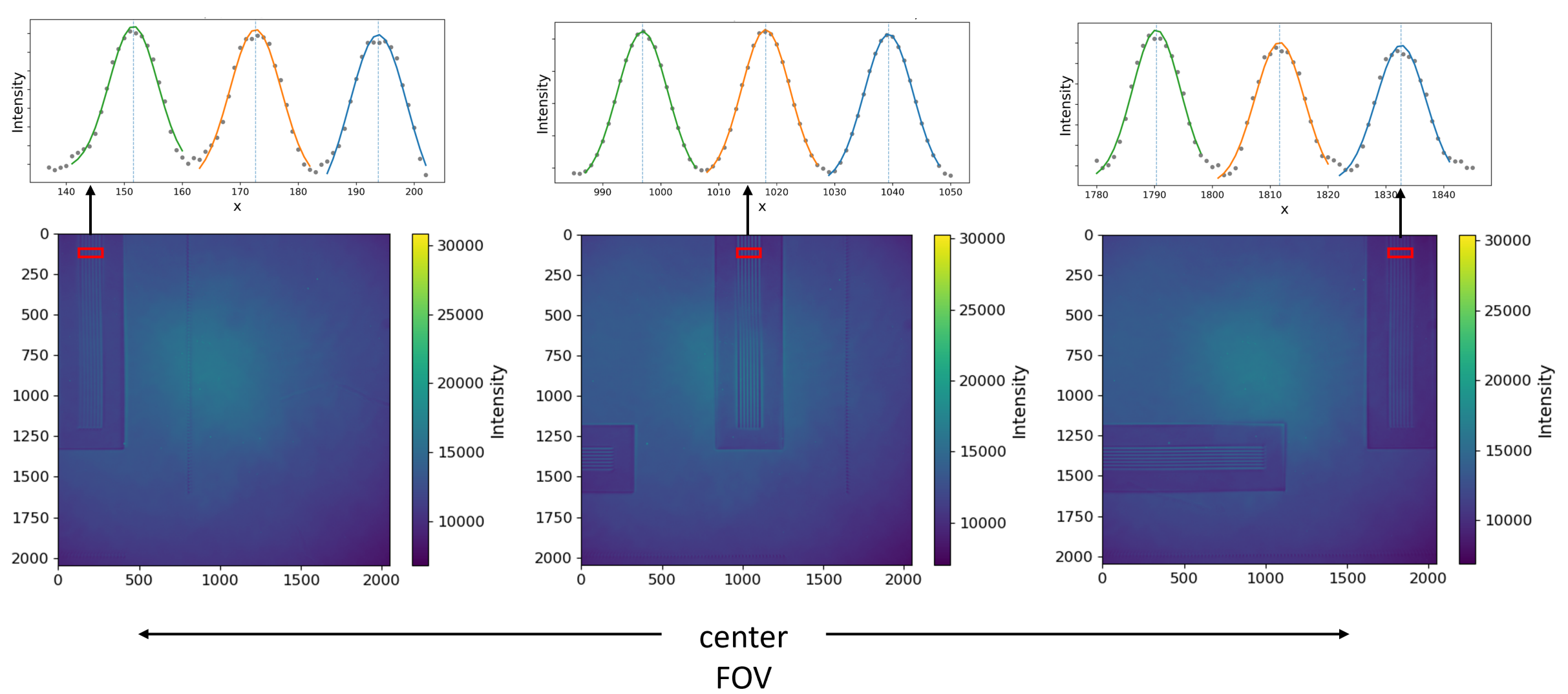

Figure S7: Strategy used to estimate the blur in the FOV due to the use of pink illumination. For each image of both the monochromatic and pink datasets, three central oscillations representing the pillars of the JIMA target were fitted with a Gaussian model to extrapolate the fwhm, which corresponds to the width of the pillars. The analysis was done at the top part of the FOV as shown here and at the center.

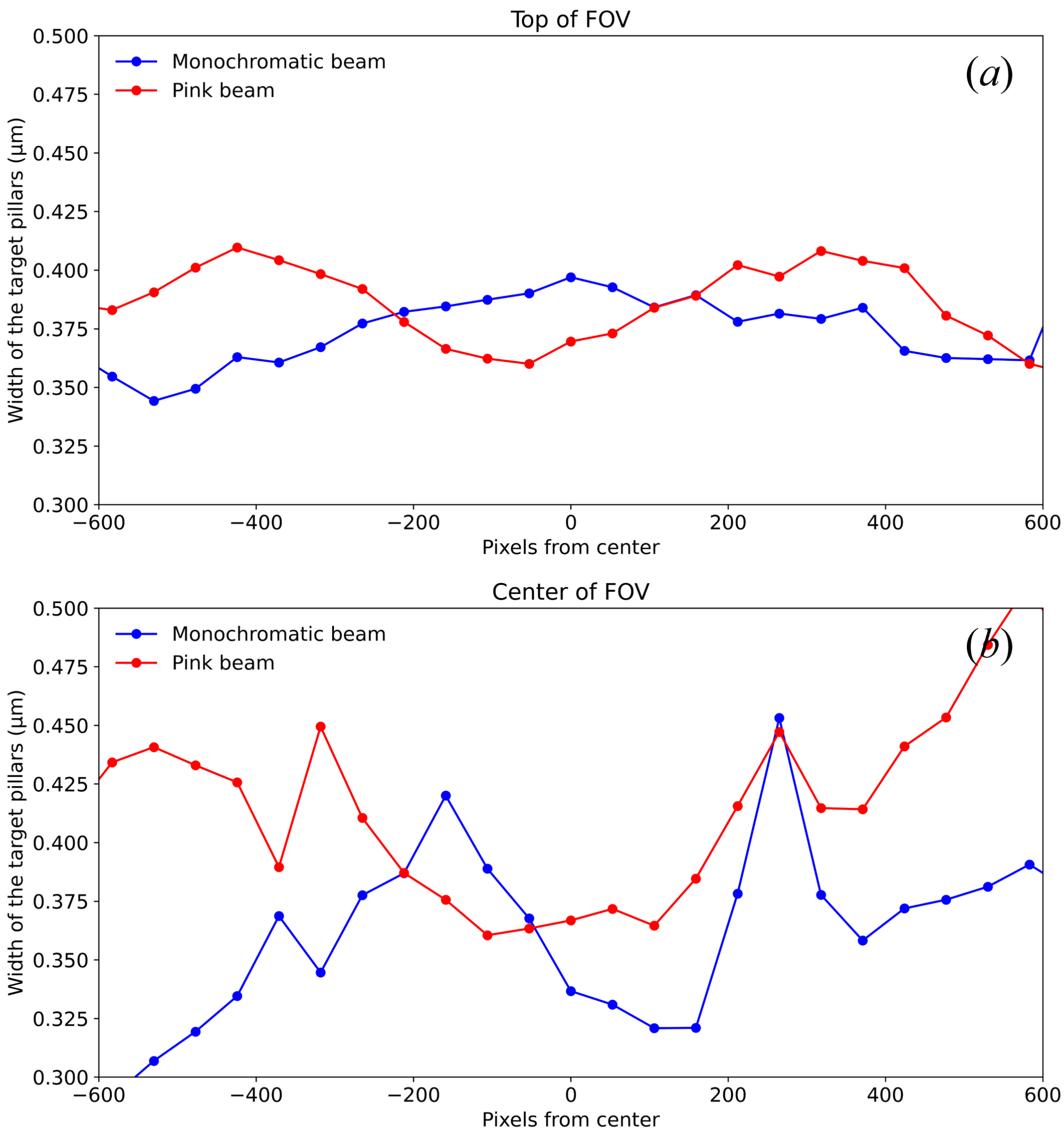

Figure S8: Results of the fitting procedure explained in Fig.S4. The mean FWHM calculated from the three oscillations in each image was transformed in μm knowing the effective pixel size of the datasets. a) Results for the top part of the FOV (y=100). b) results for the center part of the FOV (y=1000).

ideal contrast, respectively. Fig.S7 shows the Michelson contrast as a function of the distance from the center of the FOV for monochromatic and pink beam illumination. The datasets are the same reported in Fig.S5. The monochromatic beam yields higher contrast across the FOV, indicating that the sharpness of the image is better preserved than in the pink beam case.

Quantification of the blur: To quantify the blur associated with the pink beam illumination, we have fitted the oscillations of the JIMA target across the full horizontal range at the top and at the center of the FOV. The strategy used is summarized in Fig.S7. For each image, we extracted a horizontal profile averaging over 10 pixels to improve the SNR. Then, we selected three oscillations from the center of the JIMA target, and we fitted each one of them independently with a Gaussian model. For each image, we calculated the mean FWHM of the three oscillations, and knowing the effective pixel size, we calculated the width corresponding to the JIMA pillars in μm. The results are reported in Fig.S8 for the top (a) and central part of the FOV (b). The values coming from the Gaussian fitting for the monoclinic and pink datasets are quite close to each other in both areas of the FOV. This suggests that the use of a pink beam in this case did not introduce evident blur on direct space images in bright field conditions.

5 Dark-field ray-tracing through a CRL

We present an analytical and numerical framework for simulating diffracted x-ray beams in compound refractive lens systems (CRL) used in Dark-Field X-ray Microscopy (DFXM and pDFXM). The direct beam divergence- and wavelength-statistics are modulated through sample interaction using the Laue equations with a kinematical scattering approximation. Using the resulting diffracted beam statistics, rays are repeatedly sampled and traced through the objective system to render a virtual 2D detector image of a crystalline sample (i.e the phantom). In our framework, rays are parameterized by a 3D spatial coordinate, a propagation direction (unit vector), and, importantly, a unique photon energy. The latter gives rise to ray-dependent attenuation via Beer's law, as well as ray-dependent refraction. Free-space propagation together with parabolic-lens refraction are handled via explicit, nonlinear ray-transfer functions, going beyond paraxial ABCD matrices by explicitly solving Snell's law at every lens interface. Monte Carlo sampling is used to propagates ensembles of rays.

5.1 Ray Formalism

We work in the cartesian imaging coordinate system $(\hat{\boldsymbol{x}}_i, \hat{\boldsymbol{y}}_i, \hat{\boldsymbol{z}}_i)$ with the x-axis along the optical axis of the *diffracted* beam, the z-axis pointing upward (toward the roof), and the y-axis transverse. The CRL lens-lets are assumed to be centered on the optical axis, perfectly aligned, and rotational symmetric with respect to the optical axis.

In our formulation, a ray is primarily represented by six scalars grouped into two vectors. The (normalized) direction of propagation is given by $\hat{\boldsymbol{r}} \in \mathbb{R}^3$ while the spatial position of the ray is given by $\boldsymbol{x} \in \mathbb{R}^3$.

Two describe the optical system at hand it is enough to consider

1. Free-space propagation of a ray, and,

2. Propagation through a parabolic lens.

The traversed path of a ray scattering off the sample can then be described as (i) a free-space propagation to the CRL entry, (ii) a series of interlaced lens-propagations and (shorter) free-space propagations, as the ray makes it though the objective, and, finally (iii) a free-space propagation from the CRL exit to the detector surface. At each propagation step we subscript ray-properties before the propagation with j and ray resulting ray-properties, after propagating through the element, with $j+1$.

5.1.1 Free-Space Propagation

In free space, the ray direction is unchanged,

$$\hat{\boldsymbol{r}}_{j+1} = \hat{\boldsymbol{r}}_j, \tag{3}$$

while the ray position is updated linearly with the propagation distance L as

$$\boldsymbol{x}_{j+1} = \boldsymbol{x}_j + \left(\frac{L}{\hat{\boldsymbol{x}}_i^T \hat{\boldsymbol{r}}}\right)\hat{\boldsymbol{r}}. \tag{4}$$

5.1.2 Parabolic Concave Lens

For x-rays with refractive index $n = 1 - \delta$ ($\delta > 0$), we now seek to derive the propagation path through a parabolic concave lens. The web thickness is $T_{web} = h_2 - h_1$ at the apex with $x = h_1$ at the apex entry and $x = h_2$ at the apex exit ($h_2 > h_1$, see also Figure S9). For such curved lenses the outgoing ray position and direction are coupled functions of the ingoing ray position and direction. The parabolic entry and exit surfaces are defined as

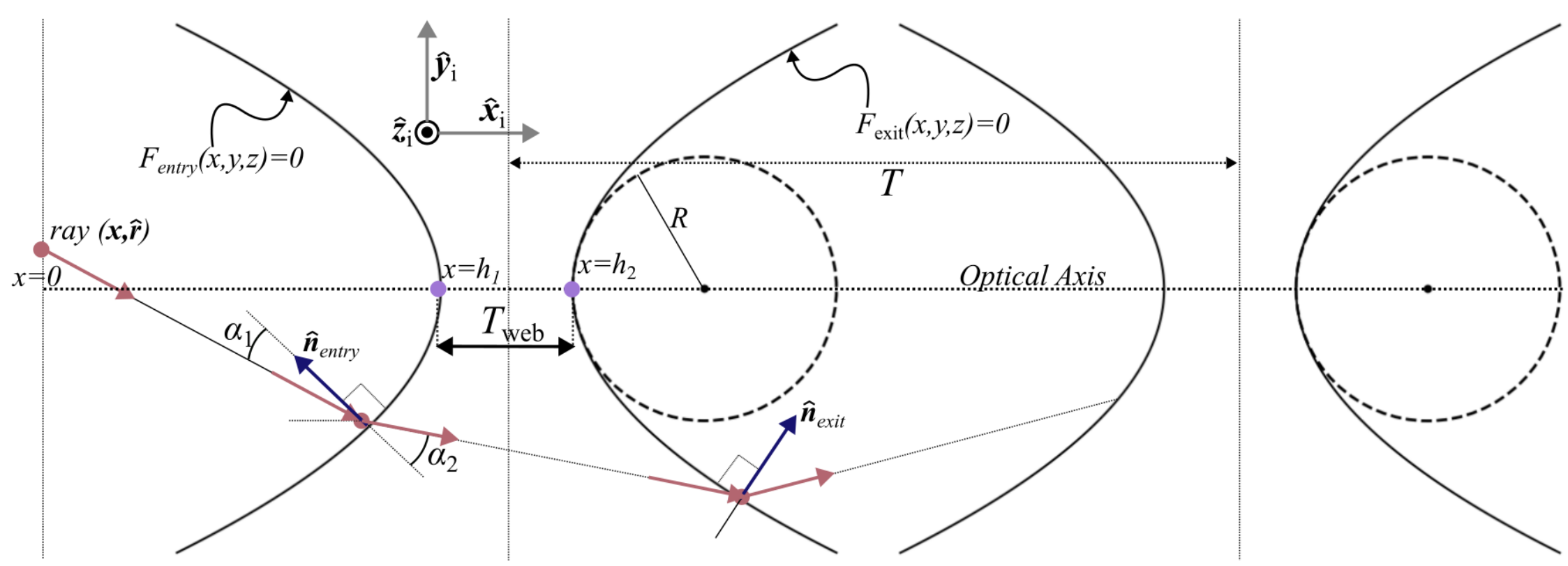

Figure S9: Parabolic series of lenses with a traveling ray. The lens surfaces are described as analytical level sets, which provide closed-form expressions for the surface normals. Each parabola has curvature R at the apex and a web thickness T_{web}, with lens center-to-center spacing T. The lenses are rotational symmetric around the optical axis with apex normal aligned with $\hat{\boldsymbol{x}}_i$.

$$F_{entry}(\boldsymbol{x}) = x - h_1 + \left(\frac{y^2 + z^2}{2R}\right) = 0, \tag{5}$$

$$F_{exit}(\boldsymbol{x}) = x - h_2 - \left(\frac{y^2 + z^2}{2R}\right) = 0. \tag{6}$$

Their normals are given by

$$\hat{\boldsymbol{n}}_{entry}(x, y, z) = p_0 \begin{bmatrix} R & y & z \end{bmatrix}^T, \tag{7}$$

$$\hat{\boldsymbol{n}}_{exit}(x, y, z) = p_0 \begin{bmatrix} R & -y & -z \end{bmatrix}^T, \tag{8}$$

with normalization

$$p_0 = \frac{1}{\sqrt{R^2 + y^2 + z^2}}. \tag{9}$$

The ray intersects the entry surface at

$$F_{entry}(\boldsymbol{x} + \hat{\boldsymbol{r}}\, s_{in}) = 0, \tag{10}$$

which expands in vector form as

$$F_{entry}(\boldsymbol{x}) = \boldsymbol{x}^T \hat{\boldsymbol{x}}_i - h_1 + \left(\frac{\boldsymbol{x}^T \boldsymbol{x} - (\boldsymbol{x}^T \hat{\boldsymbol{x}}_i)^2}{2R}\right). \tag{11}$$

Inserting the parametric ray $(\boldsymbol{x} + \hat{\boldsymbol{r}}\, s_{in})$ yields a quadratic equation for the intersection distance s_{in}:

$$\begin{aligned} &F(\boldsymbol{x}) = 2R(\boldsymbol{x}^T \hat{\boldsymbol{x}}_i + \hat{\boldsymbol{r}}^T \hat{\boldsymbol{x}}_i s_{in} - h_1) + \\ &\Big(\boldsymbol{x}^T \boldsymbol{x} + 2\hat{\boldsymbol{r}}^T \boldsymbol{x} s_{in} + s_{in}^2 - (\boldsymbol{x}^T \hat{\boldsymbol{x}}_i)^2 - 2(\boldsymbol{x}^T \hat{\boldsymbol{x}}_i) \\ &(\hat{\boldsymbol{r}}^T \hat{\boldsymbol{x}}_i s_{in}) - (\hat{\boldsymbol{r}}^T \hat{\boldsymbol{x}}_i)^2 s_{in}^2\Big) = 0. \end{aligned} \tag{12}$$

Introducing the scalars

$$\begin{aligned} a &= \boldsymbol{x}^T \boldsymbol{x}_i, \quad b = \hat{\boldsymbol{r}}^T \boldsymbol{x}_i, \\ c &= \boldsymbol{x}^T \boldsymbol{x}, , \quad d = \hat{\boldsymbol{r}}^T \boldsymbol{x}, \end{aligned} \tag{13}$$

we obtain

$$\begin{aligned} &F(\boldsymbol{x}) = 2R(a + b s_{in} - h_1) + \\ &\left(c + 2d s_{in} + s_{in}^2 - a^2 - 2ab s_{in} - b^2 s_{in}^2\right) = 0. \end{aligned} \tag{14}$$

The exact quadratic solution is

$$s_{in} = \frac{Rb - ab + d - \sqrt{\kappa(h_1) + \beta}}{b^2 - 1}, \tag{15}$$

with

$$\begin{aligned} \kappa(h_1) &= 2R(-a - b^2 h_1 + bd + h_1), \\ \beta &= R^2 b^2 + a^2 - 2abd + b^2 c - c + d^2. \end{aligned} \tag{16}$$

Introducing a local coordinate system around the current ray location $\boldsymbol{x}$ such that $a = c = d = 0$ and setting $\Delta h = h - x$ we find by Taylor expanding equation (15) around $b \approx 1$ that:

$$s_{in} \approx \Delta h(q_2 - q_1) \tag{17}$$

with $q_1 = (1-b)/R$ and $q_2 = (2-b)$. This approximation holds for small to moderate values of $\epsilon = |1-b|$. This can be though of as an intermediate paraxial approximation that stabilizes numerical precision by avoiding (close to) singular division in (15). Without these numerical considerations, we found accumulated errors to become prohibitively large for lens-trains that features more than 10 lenses (using float64 representations throughout). Hence, the use of local coordinates, and Taylor expansion of (15), is key for our framework, which features a CRL with 87 lens-lets.

Having located the ray entry point, the angular shift is computed from Snell's law. The incidence angle is

$$\sin(\alpha_1) = ||\hat{\boldsymbol{n}}_{entry} \times \hat{\boldsymbol{r}}||_2, \tag{18}$$

and the refracted angle is

$$\alpha_2 = \arcsin\left(\frac{\sin(\alpha_1)}{1-\delta}\right). \tag{19}$$

Letting

$$\boldsymbol{k} = \frac{\hat{\boldsymbol{n}}_{entry} \times \hat{\boldsymbol{r}}}{||\hat{\boldsymbol{n}}_{entry} \times \hat{\boldsymbol{r}}||_2}, \tag{20}$$

the updated ray direction follows from the Rodriguez rotation formula:

$$\begin{aligned}\hat{\boldsymbol{r}} &= \hat{\boldsymbol{n}}\cos(\alpha_2) + (\boldsymbol{k} \times \hat{\boldsymbol{n}})\sin(\alpha_2) \\ &+ \boldsymbol{k}(\boldsymbol{k}^T\hat{\boldsymbol{n}})(1-\cos(\alpha_2)).\end{aligned} \tag{21}$$

Propagation inside the lens is determined by solving again for the intersection with the exit surface. Applying the same trick of local coordinates and Taylor expansion we find that

$$s_{out} \approx \Delta h(q_2 + q_1) \tag{22}$$

with $q_1 = (1-b)/R$ and $q_2 = (2-b)$. Note that the scalar s_{out} corresponds to the path length through the medium. The corresponding attenuation therefore applies to the ray:

$$I = I_0 \exp(-\mu s_{out}). \tag{23}$$

Finally, we apply the Snell/Rodriguez update of equations (18)–(21) at the exit surface using the appropriate normal vector to obtain the outgoing direction.

Both the linear attenuation coefficient, μ, and the refractive decrement, δ, are wavelength dependent quantities. While μ and δ are assumed to remain constant for a single ray, throughout propagation, they must be taken to vary between different scattered rays. The seeding values of μ and δ, together with the initial propagation direction of the ray are computed by letting the direct beam interact with the crystalline sample through the Laue equations.

5.1.3 Laue Conditions

To simulate diffraction from the sample microstructure we start by generating a large *canonical* pool of N_0 incident directions, modeling the horizontal and vertical divergences of the direct x-ray beam as two independent Gaussian distributions,

$$\xi_h \sim \mathcal{N}\big(0, \sigma_{\xi,h}^2\big)\,, \qquad \xi_v \sim \mathcal{N}\big(0, \sigma_{\xi,v}^2\big)\,. \tag{24}$$

The source spectrum defines a prior on energy distribution in the direct beam as:

$$E \sim \mathcal{N}\big(\bar{E},\, \sigma_E^2\big)\,. \tag{25}$$

Using the Laue equations the scattering direction of each incident ray can now be calculated in lab-coordinates as

$$\hat{\boldsymbol{n}}_l = \hat{\boldsymbol{n}}_l^{direct} - 2(\hat{\boldsymbol{x}}_l^T \hat{\boldsymbol{q}}_l)\hat{\boldsymbol{q}}_l \tag{26}$$

where $\hat{\boldsymbol{q}}_l$ is the lattice normal in lab frame, $\hat{\boldsymbol{x}}_l$ is the mean beam propagation direction, and $\hat{\boldsymbol{n}}_l^{direct}$ is a sampled incident ray from the direct beam following equations (24). Th scattered ray direction, $\hat{\boldsymbol{n}}_l$, can then be brought into the imaging system by applying a fixed rotation corresponding to the mean Bragg angle.

The Bragg geometry at the sample fixes the wavelength for each candidate direction via

$$E_i^{\mathrm{req}} = \frac{hc}{\lambda_i} = \frac{hc}{2d\sin\theta_i}, \tag{27}$$

where h is planck's constant and c the speed of light. I.e, only if the energy is exactly E_i^{req} will scattering occur. This is modeled as weighting the ray intensity by the likelihood of E_i^{req} as prescribed by the direct beam energy distribution.

5.2 Monte Carlo Framework

The crystalline phantom sample is discretised into voxels taken to lie in the sample plane (i.e a 2D grid of 3D voxels that sits on the goniometer). For each occupied voxel in the object plane we launch a fixed number of rays with uniform sub-voxel jitter in the transverse directions,

$$\Delta y \sim \mathcal{U}\Big(-\tfrac{d_y}{2}, \tfrac{d_y}{2}\Big)\,, \qquad \Delta z \sim \mathcal{U}\big(-\tfrac{d_z}{2}, \tfrac{d_z}{2}\big)\,, \tag{28}$$

where d_y and d_z are the voxel sizes along the imaging y- and z-axes, respectively. The ray launch point is

$$\boldsymbol{x}_{\mathrm{launch}} = \boldsymbol{x}_0 + (0,\, \Delta y,\, \Delta z), \tag{29}$$

with $\boldsymbol{x}_0$ the voxel center. Each ray inherits its initial photon intensity from the object material density at the ray launch point multiplied with the spectral likelihood, $p(E_i^{\mathrm{req}})$. The propagation direction of the diffracted ray, $\hat{\boldsymbol{n}}$, is computed from the direct beam statistics and the Laue equations as described previously in section 5.1.3. Likewise, the diffracted energy, E_i^{req}, of the ray is computed from the Bragg equations. In practice we here perform a stratified systematic resample of the pool using the spectral prior evaluated at E_i^{req} to concentrate on Bragg-compatible directions and not waste the majority of floating point

operations on near zero intensity rays. I.e we focus the sampling of the direct beam to rays that have a non-zero likelihood of actually scattering off the sample.

Rays are then transported through the optical train by chaining the nonlinear transfer functions described in sections 5.1.1-5.1.2. Energy-dependent refraction $(1 - \delta(E))$ and absorption $(\mu(E))$ is simulated per ray. Representative values for Beryllium were collected form the NIST database.

As the rays arrive at the detector surface we bin each ray to the nearest pixel and deposit the respective intensity. Accumulating many rays in this way, a magnified (and inverted) 2D image of the phantom sample is formed. Since each ray corresponds to an independent path through the microscope system, the algorihtm is embarrassingly parallel. Transport is executed on the GPU using a batched kernel that implementing the analytic intersections and refraction updates described above. The algorithm scales linearly with the number of lens surfaces and can supports millions to hundreds of millions of rays per image. Using a NVIDIA RTX 1000 Ada Generation GPU (6141 MiB VRAM, CUDA 12.4, driver 550.144.03), for a system featuring 87 lens-lets, using a 64-point floating point precision, we observed benchmarks of 1.2×10^6 traced rays per seconds.

References

[1] Y-H. Chu, M. P. Cruz, C-H. Yang, L. M. Martin, P-L.Yang, J-X. Zhang, K. Lee, P. Yu, L-Q. Chen, and R. Ramesh. Domain control in multiferroic bifeo3 through substrate vicinality. *J Adv Mater*, 19:2662–2666, 2007. doi: 10.1002/adma.200602972.

[2] Hugh Simons, Anders Clemen Jakobsen, Sonja Rosenlund Ahl, Henning Friis Poulsen, Wolfgang Pantleon, Ying-Hao Chu, Carsten Detlefs, and Nagarajan Valanoor. Nondestructive mapping of long-range dislocation strain fields in an epitaxial complex metal oxide. *Nano Lett.*, 19:1445–1450, 2019. doi: 10.1021/acs.nanolett.8b03839.

[3] Henning Friis Poulsen, Anders Clemen Jakobsen, Hugh Simons, Sonja Rosenlund Ahl, Philip K. Cook, and Carsten Detlefs. X-ray diffraction microscopy based on refractive optics. *J. Appl. Cryst.*, 50:1441, 2017. doi: 10.1107/S1600576717011037.

[4] Can Yildirim, Aditya Shukla, Yubin Zhang, Nikolas Mavrikakis, Louis Lesage, Virginia Sanna, Marilyn Sarkis, Yaozhu Li, Michela La Bella, Carsten Detlefs, et al. Pink-beam dark field x-ray microscopy: Expanding 3d/4d imaging for complex and deformed microstructures. *arXiv preprint arXiv:2503.05921*, 2025.

[5] Júlia Garriga Ferrer, Raquel Rodríguez-Lamas, Henri Payno, Wout De Nolf, Phil Cook, Vicente Armando Solé Jover, Can Yildirim, and Carsten Detlefs. darfix – data analysis for dark-field X-ray microscopy. *Journal of Synchrotron Radiation*, 30:527–537, 2023. doi: 10.1107/S1600577523001674. URL `https://journals.iucr.org/paper?gy5041`.
